# Aerogels of Chitosan–Pectin–Lactic Acid Loaded with MOFs: Performance and Kinetics in Removal of Dyes

**DOI:** 10.3390/polym17152008

**Published:** 2025-07-23

**Authors:** Tomás Soteras, Ignacio Manuel Argento Arruñada, Leila María Saleh Medina, Natalie Malikova, Koro de la Caba, Pedro Guerrero, Norma Beatriz D’Accorso, R. Martín Negri

**Affiliations:** 1 Departamento de Química Inorgánica, Analítica y Química Física, Facultad de Ciencias Exactas y Naturales, Universidad de Buenos Aires, Ciudad Universitaria, Pabellón 2, Ciudad Autónoma de Buenos Aires C1428EGA, Argentina; soteras@qi.fcen.uba.ar (T.S.); lsmedina@qi.fcen.uba.ar (L.M.S.M.); 2 Instituto de Química Física de Materiales, Ambiente y Energía (INQUIMAE), Consejo Nacional de Investigaciones Científicas y Técnicas (CONICET)-Universidad de Buenos Aires (UBA), Ciudad Universitaria, Pabellón 2, Ciudad Autónoma de Buenos Aires C1428EGA, Argentina; 3 Departamento de Química Orgánica, Facultad de Ciencias Exactas y Naturales, Universidad de Buenos Aires, Ciudad Universitaria, Pabellón 2, Ciudad Autónoma de Buenos Aires C1428EGA, Argentina; 4 Centro de Investigaciones en Hidratos de Carbono (CIHIDECAR), Consejo Nacional de Investigaciones Científicas y Técnicas (CONICET)-Universidad de Buenos Aires (UBA), Ciudad Universitaria, Pabellón 2, Ciudad Autónoma de Buenos Aires C1428EGA, Argentina; 5 Laboratory of Physical Chemistry of Electrolytes and Interfacial Nanosystems (PHENIX), Sorbonne Université, CNRS, 75252 Paris, France; natalie.malikova@sorbonne-universite.fr; 6 BIOMAT Research Group, University of the Basque Country (UPV/EHU), Escuela de Ingeniería de Gipuzkoa, Europa Plaza 1, 20018 Donostia-San Sebastián, Spain; koro.delacaba@ehu.es

**Keywords:** bio-based aerogels, MOFs, removal kinetics

## Abstract

Aerogel sponges of bio-based polymers loaded with metal–organic frameworks (MOFs) are highly promising for environmental applications, but a central challenge is to improve their stability and efficiency for removal processes. Here, the effective incorporation of the MOFs MIL-100(Fe) and ZIF-8 in composite aerogels of chitosan–pectin–lactic acid is reported. The presence of pectin was critical to loading the MOFs efficiently and homogeneously, while the incorporation of lactic acid induced a large increase in the Young’s modulus and provided structural preservation in aqueous solutions. The presence of MOFs enhanced the removal of two dyes, methyl orange (MO) and methylene blue (MB), under batch and flow conditions, with removal efficiencies of methyl orange of about 85% and 90% when loaded with ZIF-8 and MIL-100(Fe), respectively. Bentonite, celite 545, and two ionenes were loaded for comparison. Factors beyond charge-to-charge electrostatic interactions influenced the removal, since no correlations were obtained between the electrical charges of dyes, fillers, and polymers. The kinetic data were analyzed by adapting the Langmuir kinetic model, incorporating absorption and desorption processes, which allowed the recovery of the respective rate constants.

## 1. Introduction

The use of metal–organic frameworks (MOFs) as fillers incorporated into matrices for environmental applications in liquid media, such as the removal of dyes and heavy metals from aqueous solutions, has received great interest due to their high porosity and surface areas and the possibility of obtaining very diverse structures with variable surface charges and wettability [1,2,3,4,5,6,7,8,9,10]. Incorporating MOFs into hydrophilic biopolymers has gained increasing relevance [1,11,12,13,14,15,16], since these matrices constitute environmentally friendly alternatives to synthetic polymers for removal and separation processes [17,18,19,20,21,22,23,24,25,26,27,28,29,30,31,32,33,34,35,36,37]. Important efforts have been made to add MOFs into membranes and beads of commercially available biopolymers, mainly chitosan, celluloses, pectins, and alginates [38,39,40,41,42,43,44,45].

In particular, aerogel sponges made by lyophilization of biopolymer hydrogels provide dry, spongy, and highly ductile materials [46,47,48,49,50,51,52,53,54]. However, aerogel sponges loaded with MOFs have been scarcely explored, particularly concerning the removal of pollutant species or oil–water separation processes [55,56,57,58,59,60,61,62]. One critical factor in lyophilized sponges is the incorporation of MOF crystals into the biomaterial, ensuring the satisfaction of several requirements such as high loading efficiency in relatively short preparation times, achieving a spatial distribution that is as homogeneous as possible and without subsequent separation of the matrix–filler phases. In connection with this issue, two of the present authors have reported the effective incorporation of UiO66 MOFs into freeze-dried chitosan sponges by adding pectin [12]. This system has been scarcely studied; few studies of the physical–chemical properties of chitosan–pectin dispersions and membranes have been reported, and even fewer studies on lyophilized chitosan–pectin sponges exist in the literature [12,63]. Moreover, there is a lack of systematic studies concerning the sorption kinetics (absorption and/or adsorption) of analytes and the possibility of improving the merit parameters in removal processes by adding MOFs in lyophilized chitosan–pectin–MOF composite sponges. Therefore, several issues related to the characteristics of these aerogels in water remediation processes, like the relevant environmental case of dye removal, are still unclear [64,65,66] and are related to several questions: Is it possible to homogeneously incorporate cationic or anionic MOFs into chitosan–pectin hydrogels to obtain aerogel sponges, providing long-term stability, high sorption efficiency, and reusability for dye removal applications? On what chemical and physical bases can these properties be rationalized? In particular, what is the role of electrical charges in the sponges when they are placed in contact with aqueous solutions to be remediated?

Concerning removal kinetics, a relevant issue is associated with the discussion about whether simple kinetic models based on first- or second-order kinetics may describe, and if so how, the uptake of dyes in batch experiments. It is worth noting that removal kinetics are usually described by pseudo-second-order models only on the base of an empirical approximation to data fitting. For instance, recently, İpek et al. [67] studied the removal of the dye Basic Yellow by chitosan particles, reporting that a pseudo-second-order kinetic model best describes the removal process, and Rahul and Jindal [68] recently reported second-order kinetics for the removal of malachite green and fuchsin acid using cellulose-modified hydrogels. However, in systems like aerogel sponges, which present large and interconnected cavities with open structures that allow, through swelling, the easy entry and exit of the external aqueous solution with the analyte (particularly in media with a low ionic strength, where swelling is relevant), it is unlikely that processes such as desorption, which may generate a kinetic behavior that cannot be described by pseudo-second- or -first-order models, can be disregarded.

Thus, the objective of the present work is to provide a partial contribution to answering the raised questions. Central aspects are referred to the specific bio-based sponge considered here, the inclusion of additives with determined functionalities, the development of a protocol for improving the incorporation of MOFs, and the characterization of the final composite sponge. The implemented procedure and considered biopolymers allow the effective incorporation of two MOFs with different surface charges: MIL-100(Fe) (anionic) and ZIF-8 (cationic). MIL-100(Fe) and ZIF-8 are two of the most common MOFs because of their relatively simple synthesis, their stability at room temperature, and their high surface areas. However, they present important differences in properties associated with the absorption of analytes, which makes it relevant to compare their performances in removal experiments. For instance, MIL-100(Fe) is synthesized by combining coordinated iron (Fe(III)) trimers and trimesate ligands, obtaining a super-tetrahedron structure. This structure generates a bimodal distribution of cavities, referred to as cages: one set of mesoporous cages with diameters in the order of 2.5–3.3 nm, and another set of cages with diameters of about 0.5–1.0 nm, which are both of central relevance for the absorption of analytes. Note that the largest mesoporous cages are appropriate for incorporating organic dyes such as MB and MO. Two additional factors must be taken into account, both contributing to the high performance of MIL-100(Fe) in removal processes: the surface charge of the formed octahedron-shaped particles and the microporosity of the structure. The microporous and mesoporous structures generate large surface areas (~1000 m^2^/g for N_2_). Concerning the surface charges, it is known that Fe-Lewis acid sites are generated by the elimination of coordinated water molecules from the octahedral iron structure. These sites and the presence of surface carboxylic groups contribute to the acidic character of the MOF, which presents a point of zero charge at a pH of about 4.5; hence, the particles of MIL-100(Fe) present negative surface charges at a pH above 5.0. Summarizing, the main reasons for choosing MIL-100(Fe) as an absorber of organic dyes are the three mentioned factors: the presence of mesoporous cages, the micropores which contribute to a large surface area, and the net negative surface charge at the pH of work in removal experiments (pH ≅ 6.0). Although ZIF-8 has a large surface area, as MIL-100(Fe), there are several differences between the other properties related to sorption studies when comparing ZIF-8 and MIL-100(Fe). First, ZIF-8 and MIL-100(Fe) present surface electrical charges of opposite signs: ZIF-8 does not present (-COO^−^) groups, and the exposed metal on the external surface, Zn^2+^, is coordinated to the 2-methylimidazole groups, not to water molecules. Thus, the net electrical surface charge of the most common particle morphologies of ZIF-8 (cubic, rhombic, and dodecahedral) is positive at pH = 6, since the pH of zero net charge is about 9. Second, ZIF-8 does not present mesoporous cages as MIL-100(Fe); thus, ZIF-8 is classified as a microporous material. These considerations suggest that, in principle, ZIF-8 might have a lower removal capacity than MIL-100(Fe) and be differentiated according to the net charge of the analyte. Therefore, the reasons for the selection of these MOFs are their similarities (stability and high surface areas) and differences (surface charges of opposite signs and different porosities).

Although MOFs are promising compounds for sorbent applications, they may present some drawbacks in comparison with other absorbents related to their lower thermal stability and costs. It is also relevant for comparison to explore other absorbent fillers incorporated into the sponges. Bentonite and celite 545 are clays usually employed as inexpensive and safe sorbents; they possess negative surface charges, stable structures, mechanical and thermal stability, and high specific surface areas [69,70]. Ionenes are polymeric chains with a net positive charge in their structure, compensated by a negative counter-ion. They have been used in applications from membrane-based separations to biomedical materials [71]. Therefore, two clays with negative surface charges (bentonite and celite 545) and two cationic polymers (3,3′ and 6,6′ ionenes) were also used as fillers for comparison. The removal application was analyzed by considering two dyes, methyl orange (MO) and methylene blue (MB), which have anionic and cationic characters, respectively. The efficiencies of dye removal were compared for the different traditional fillers, and feasibility tests for flow sorption are presented. Finally, batch sorption kinetic results are critically discussed and modeled.

## 2. Materials and Methods

### 2.1. Chemicals 

All solvents and reagents were of analytical quality and used as received. Zinc nitrate hexahydrate Zn(NO_3_)_2_·6H_2_O, zinc acetate (ZnAcO; Zn(O_2_CCH_3_)_2_·2H_2_O), 2-methylimidazole (2-MeIm), and trimesic acid 95% were purchased from Sigma-Aldrich from Buenos Aires (Argentina). Chitosan was purchased from Haili Biologic Products Co. Ltd. from Qingdao (China). Methanol and lactic acid were purchased from Sintorgan from Buenos Aires (Argentina). Sodium hydroxide was purchased from Cicarelli from Santa Fe (Argentina). The dyes, methylene blue and methyl orange, were purchased from Riedel-de Haen AG Seelze–Hannover from Seelze (Germany) and Hartens from Buenos Aires (Argentina), respectively. Celite 545 was purchased from Buchs from *Benningen a. Neckar* (Germany). A commercial citrus high-methoxylated pectin (HMP) type 105 rapid set (origin: Brazil; provided by Rosenfeld, Buenos Aires, Argentina) was used as purchased. The bentonite was purchased from Rosenfeld and Marconi (Argentina). The 3,3′ and 6,6′ ionenes were synthesized by the group of Dr. N. Malikova (Sorbonne University, France) according to already published protocols [72,73].

### 2.2. Instrumentation

Powder X-ray diffractograms (PXRDs) were recorded with a Panalytical Empyrean diffractometer using Cu radiation, K_α1_ = 1.54 Å, equipped with a PIXcel^3D^ area detector, in the range of 4–60°, in the *theta/2 theta* configuration (steps: 0.026°; count time: 50 s; detection limit: 0.5–2%). The equipment was calibrated with a silicon standard. Thermograms for thermogravimetric analyses (TGA) were recorded using a TGA-51 Shimadzu instrument, and the temperature was scanned from 20 °C to 600 °C at 10 °C/min under 30 mL/min nitrogen flux. Differential scanning calorimetry (DSC) analyses were performed with a DSC Shimadzu-50 instrument in the 20–170 °C range at a scanning rate of 10 °C/min under a N_2_(g) atmosphere. Surface morphology was analyzed using a Field Emission Scanning Electron Microscope (FESEM, Zeiss, Oberkochen, Germany; Zeiss Supra 40 Gemini). A lyophilizer Christ Alpha 1–2 instrument was used for sublimation at reduced pressure (0.63 mbar).

### 2.3. Synthesis and Characterization of ZIF-8

A quantity of 2.0 g of Zn(NO_3_)_2_·6H_2_O was dissolved in 50 mL of methanol at room temperature (22–25 °C) in an Erlenmeyer flask (solution A). In parallel, 4.4 g of 2-methylimidazole (2-MeIm) was dissolved in 50 mL of methanol in another Erlenmeyer flask, also at room temperature (solution B). Then, solution A was quickly incorporated into B (8:1 molar ratio between 2-MeIm and Zn^2+^) and kept under permanent stirring (200 rpm) for a given time (1, 2, and 24 h were tested, although the results did not show a significant dependence on the MOF synthesis time). A white precipitate was separated and washed by ultracentrifugation cycles in methanol (at least three cycles of 30 min at 1800 rpm). After centrifugation, the deposited material was dried at 60 °C and then pulverized in an agate mortar. The synthesized material was characterized by PXRD, ATR-FTIR, SEM, TGA, and N_2_(g) adsorption isotherms. We have recently published the PXRD diffractogram, ATR-FTIR spectrum, N_2_(g)-adsorption isotherm, and thermogram of the synthesized ZIF-8 [3], which are in excellent agreement with results previously reported by other authors [74,75,76,77]. In particular, N_2_-BET areas, S_BET_, of about (1800 ± 200) m^2^/g were obtained by fitting the N_2_(g) adsorption isotherms, which are typical reported values for ZIF-8 [71].

### 2.4. Synthesis and Characterization of MIL-100(Fe)

A quantity of 0.912 g of NaOH was dissolved in 22.8 g of H_2_O in a 250 mL beaker. Then, 1.67 g of trimesic acid was dissolved in the solution (from now on, solution 1) under continuous stirring (200 rpm) and heating. In parallel, 2.26 g FeCl_2_·4H_2_O was dissolved in 97.2 g H_2_O in another 250 mL beaker (from now on, solution 2) kept under permanent stirring (200 rpm) and heating. Then, solution 2 was added to solution 1 at a frequency of one drop per second under permanent stirring (200 rpm) overnight at room temperature. A brown dispersed material was observed after 24 h, which was then separated and washed by ultracentrifugation cycles in water (at least three cycles of 30 min at 1800 rpm). After centrifugation, the deposited material was dried at 60 °C and then pulverized with a mortar. The PXRD diffractogram and N_2_(g)-adsorption isotherms of the synthesized MIL-100(Fe) are presented in Figure S1 (Supplementary Materials), which are coincident with those previously reported [78,79]. In particular, the N_2_-BET area, S_BET_ = (1450 ± 90) m^2^/g, was obtained by fitting the N_2_(g) adsorption isotherms and is in the range of typical reported values for MIL-100 (Fe).

### 2.5. Characterization of Pectin

The employed pectin powder had been previously characterized by nuclear magnetic resonance spectroscopy (^13^C NMR and ^1^H NMR) and Ultraviolet Matrix-Assisted Laser Desorption/Ionization Time-of-Flight Mass Spectrometry (UV-MALDI-TOF) [80]. Several matrices were tested in the experiments on UV-MALDI_TOF: b-Carboline (9H-pyrido [3,4-b]indole) norharmane, 2,5-dihydroxybenzoic acid (2,5-DHB, gentisic acid), and transa-cyano-4-hydroxycinnamic acid (CHCA). The best experimental conditions were achieved using norharmane as a matrix (5 mg of norharmane was dissolved in MeOH/H_2_O 3:2 *v*/*v*), and the negative ion mode was used. The whole procedure of sample preparation is described in detail in [80]. The degree of methylation calculated from the ratio between the anomeric carbon signals is 68%, while the analogous procedure for C-6 resonances provides a degree of 60%. The average molecular weight is about 6 kDa.

### 2.6. Characterization of Chitosan 

The degree of deacetylation, obtained by acid–base back-titration [81], is 90%. The average molecular weight (*M_v_*), determined by viscosimetry, is (345 ± 10) kDa, applying the Mark–Houwink equation as reported by Kasaai et al. [82].

### 2.7. Preparation of the Lyophilized Sponges 

The following protocol was developed and implemented to satisfy two conditions: (i) to stabilize the dispersion of MOFs in the hydrogel before freeze-drying, avoiding separation–precipitation of the MOFs, and (ii) to avoid using volatile acids (such as acetic acid) for dispersing chitosan, whose presence could be harmful or detrimental to the freeze-drying equipment. These two requirements were achieved by incorporating citric pectin, which provides both the acidic medium required for the good dissolution of chitosan and the thickening effect that prevents the disintegration of the hydrogel and precipitation of the absorbents. The separation of the fillers from the initially formed hydrogel, followed by its precipitation, was observed in the absence of pectin and was particularly critical in the case of MOFs. Therefore, the preparation sequence described in the following sentences was adapted from a former protocol reported by two co-authors [12]. First, 250 mg of citric pectin powder (PEC) was dissolved in 25 mL of DI water (pH = 6.0) at 70 °C under stirring until a homogeneous solution was obtained. After the dissolution of PEC, the solution was left to cool to 25 °C. The pH of the pectin solution was around 5.5. Then, 160 µL (0.192 g) of lactic acid 87% (density ≅ 1.2 g/mL) was added with a micropipette, followed by the addition of 0.0769 g of the specific filler (MOFs, clays, or ionenes), under stirring. Then, 250 mg of chitosan powder (CS) was incorporated and completely dissolved in the media. Considering that equal masses of PEC and CS are included in the formulation and that the molar mass of the repetitive units is similar for both polymers (170–190 mol/g), the number of moles of the repetitive units is similar, about 10^−3^ moles for each polymer. The elemental analysis of the sponges indicated the following weight percentages of carbon, nitrogen, oxygen, and hydrogen: 38% C, 2% N, 53% O, and 7% H, which are consistent with the mentioned estimation of the molar proportion between PEC and CS, considering that the system presents lactic acid also. The weight percentages of the polymers and plasticizers (without considering water) were the following: PEC (32.5%), CS (32.5%), lactic acid (25%), and absorbent (10%). The pH of this hydrogel was 4.5. This mixture, although it was more viscous than the individual dispersions, was magnetically stirred to homogenize it and then transferred into plastic 12-well plates (diameter: 2.5 cm; height: 2.0 cm) and placed in a fridge at −15 °C for 24 h (Figure S2, Supplementary Materials). The plate with the frozen samples was immersed in liquid nitrogen for 1 min and afterwards placed in vacuum equipment for sublimation at 0.63 mbar for 24 h. Finally, the lyophilized sponges were removed from the wells and used in the different experiments and tests. A reference sponge (blank) without absorbents was prepared under the same protocol, using 250 mg of PEC, 250 mg of CS, and 140 µL of lactic acid.

### 2.8. Elasticity Tests (Texturometry) 

The elasticity of the base sponge, with and without the addition of lactic acid, was evaluated at room temperature using a compression test. A Microsystems TA-XT2i texturometer with a cylindrical aluminum probe (P/40, 40 mm diameter), which registers the applied force as a function of time when a sample is compressed, was used. The sponges were unidirectionally compressed from the top up to 40% of their initial thickness at a compression rate of 1 mm/s. Once 40% compression strain was reached, the force was held constant for 30 s, and afterwards the sample was decompressed. The strain, ε, was calculated for each time as the relative change in thickness. The stress, S, was calculated as the applied force divided by the geometrical transversal area of the sponge (perpendicular to the direction of the applied force). The lowest stresses and strains recorded (S_min_ and ε_min_, respectively) were discarded for data analysis, since they may have been influenced by transitory effects when contacting the sample with the probe. Therefore, data were fitted in the range of strains between 10 and 40%, according to Hooke’s Law: ∆S = E ∆ε, where ∆S = S − S_min_, ∆ε = ε − ε_min_, and E is the Young’s modulus, recovered from the fits of ∆S vs. ∆ε. The elasticity tests were performed at room temperature (22–25 °C) on three different prepared samples (replicates) for each sponge composition.

### 2.9. Method of ∆pH for the Determination of Proton Zero-Charge Point 

Following Baik and Lee [83], the pH at the point of zero net proton charge, pH_PZNPC_, is defined as a pH value at which the net proton surface charge is zero. Thus, pH_PZNPC_ indicates the pH of the electrical balance between protons and hydroxide anions, which are the main species that determined the charges on the sponges under the considered conditions. The following protocol for the determination of pH_PZNPC_ was implemented. First, 50 mL NaCl (0.01 M) solutions were prepared at different initial pH levels (referred to as pH_i_) in the range from 3 to 9, adjusted with HCl or NaOH solutions. Then, 10 mg of the sponge was added to each solution. The final pH of the solutions (pH_f_) was measured after 48 h. The pH difference, ∆pH = pH_f_ − pH_i_, was plotted versus pH_i_; thus, pH_PZNPC_ was determined at the point where ∆pH = 0. The experiment was duplicated for each sample (lyophilized sponges, chitosan, and pectin). The reported error bars correspond to the standard deviations. The tests were performed at room temperature (22–25 °C).

### 2.10. Kinetics of Dye Removal in Batches 

Sponge portions of the same mass (typically 30 mg, although they varied between 10 and 60 mg) taken from a given lyophilized sponge were added to a series of empty 12 mL glass vials. Then, 10 mL of the aqueous solution of each dye (of known concentration in the range of 10–50 ppm) was added into each vial, the time referred to as *t* = 0. The vials with the dye solution and sponge were agitated in a shaker at 130 rpm. Then, at a specific time, *t*, 3 mL of the aqueous phase was taken from a given vial and its visible absorption spectrum was recorded in the spectrophotometer, determining the dye concentration in the aqueous fraction at time *t*. Hence, the kinetic removal curve was constructed (determined by triplicate for each sponge–filler system). The function q(*t*) = (mg of dye absorbed at time *t*)/(grams of sponge), defined for a given concentration of dye, is plotted as a function of *t*. The equilibrium value of q(*t*) (reached at t → ∞) is indicated as q*_e_*. The reported error bars in the kinetic curves, the rate constant values, q(*t*)*,* and q*_e_*, correspond to the standard deviations by considering triplicate samples. The percentage of dye removal, R, defined as R = 100 (*m_o_* − *m_e_*)/*m_o_*, was calculated, where *m_e_* is the mass of dye remaining in the solution after reaching sorption equilibrium and *m_o_* is the initial mass of dye in the aqueous solution. The tests were performed at room temperature (22–25 °C) and at pH = (5.5 ± 0.5).

### 2.11. Flow Tests 

An ad hoc setup was implemented to perform feasibility tests of dye removal under flow conditions. A portion of the lyophilized sponge (30 mg) was placed inside a transparent PVC tube with a 1.27 cm diameter. The sponge portion was supported on a specially designed piece of Delrin^®^ to support it, which had a hole on the bottom that allowed the solution to flow through. The tube–sponge–holder system was placed vertically. It was connected from above to a 1 L ampoule containing the dye solution to be removed. The lower part of the tube, below the sponge, was connected to a peristaltic pump (APEMA, Buenos Aires, Argentina), which induced a 2.5 mL/min flux. The typical time for flowing was 25 min, although the solution recovered within the first five minutes was discarded. The tests were performed at room temperature (22–25 °C) and at pH = (5.5 ± 0.5).

## 3. Results and Discussion

First, the results concerning the morphological, thermal, and elastic characterization of the sponges are presented in Section 3.1. Then, the state of electrical charge of the sponges, particularly at the pH used in the removal experiments, is discussed (Section 3.2). The removal efficiency for MO and MB for sponges loaded with the different fillers is analyzed in Section 3.4 and Section 3.5, respectively. The kinetic data and the model used for its fitting are presented and discussed in Section 3.6. Finally, preliminary flow results are described in Section 3.7.

### 3.1. Morphological, Thermal, and Elastic Characterization of the Sponges

**Figure 1:**
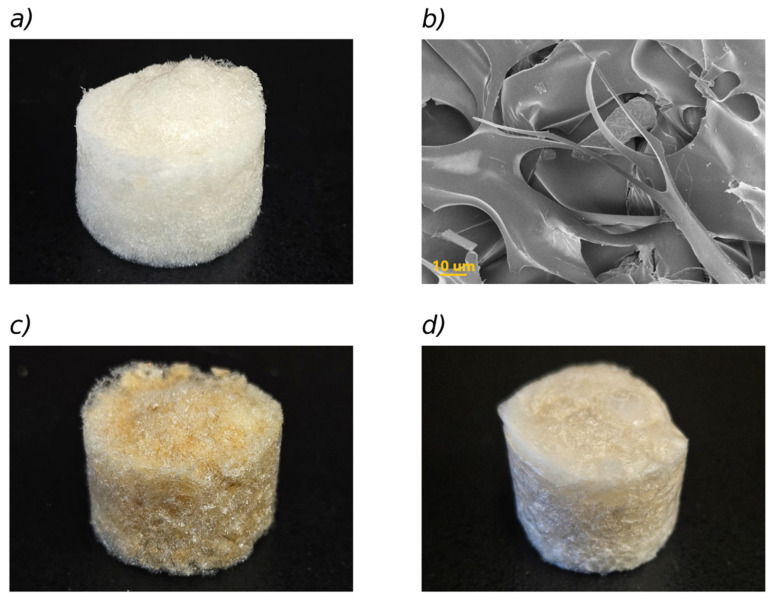
(**a**) Photograph of the base sponge (sponge without fillers). Geometrical dimensions: 1.7 cm height and 2.2 cm diameter. (**b**) SEM image of the base lyophilized sponge. (**c**) Photograph of a sponge loaded with MIL-100(Fe). (**d**) Sponge loaded with bentonite. The macroscopic dimensions are similar in (**a**,**c**,**d**).

Figure 1a shows an optical photograph of the base sponge, removed from the well after freeze-drying, while an SEM image of its interior is shown in Figure 1b.

It can be observed in the SEM image that polymeric structures in the form of twisted sheets are formed, generating open cavity-like structures with diameters of approximately 30–60 µm, which are suitable for incorporating water (swelling) when placed in contact with aqueous solutions of relatively low ionic strength. Photographs of sponges loaded with MIL-100(Fe) and bentonite are presented in Figure 1c,d, respectively, illustrating a well-defined cylindrical shape of the sponges of about 1.7 cm in height and 2.25 cm in diameter, which is conserved in the sponges with absorbents.

**Figure 2:**
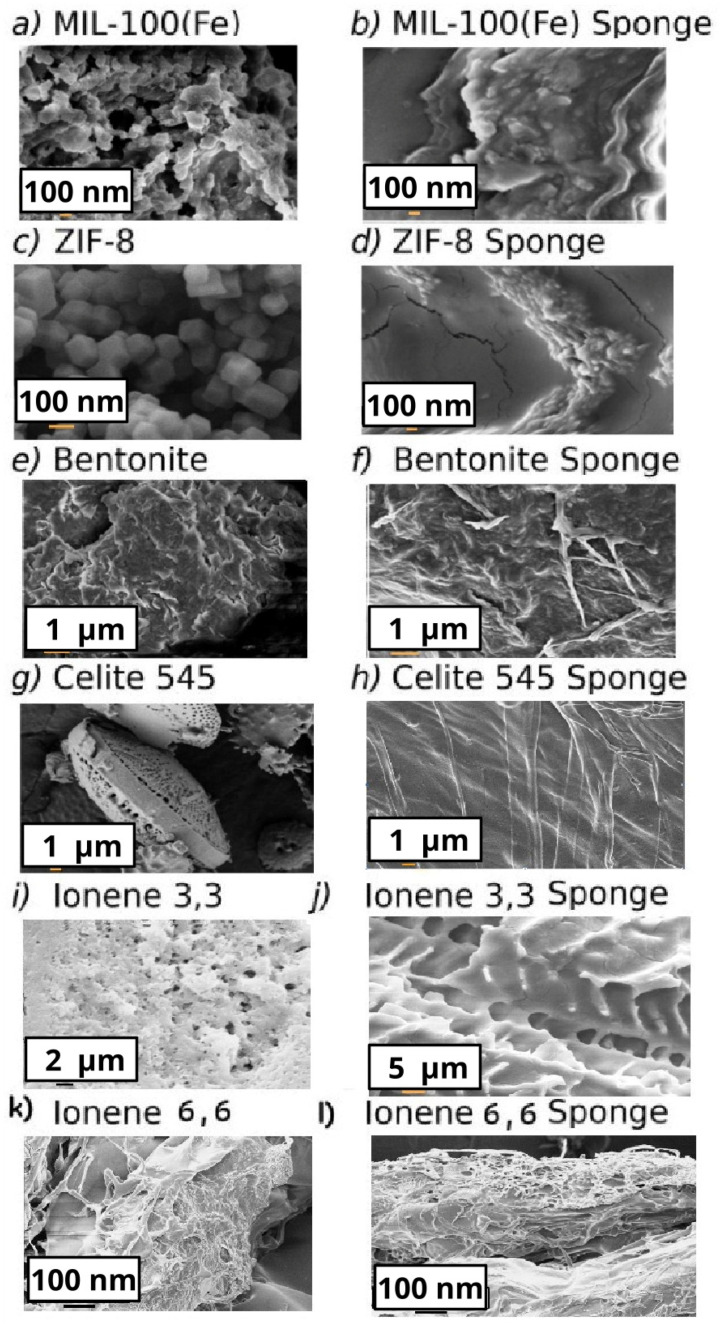
SEM images. Columns on the left: filler powder. On the right: fillers inside the sponges. The scale units on the bars are nanometers for (**a**–**d**) and micrometers for the others.

Figure 2 shows SEM images of the filler powders and the composites. The particles in the sponges appear to be coated by an amorphous structure associated with the polymers. Still, the respective nano- or micromorphologies of the fillers can be observed.

**Figure 3:**
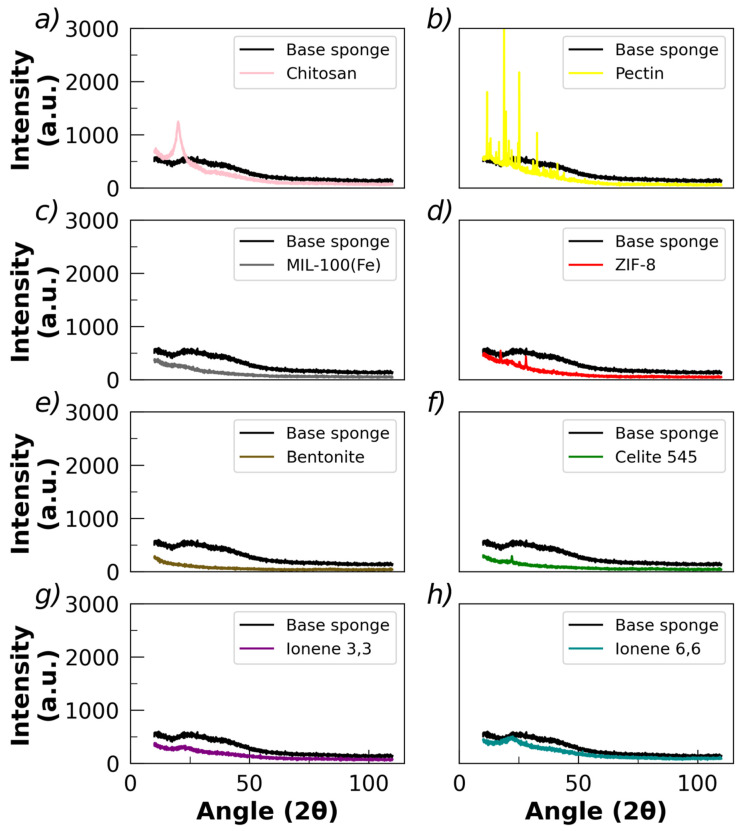
X-ray diffractograms of the powders: (**a**) chitosan and (**b**) pectin. X-ray diffractograms of the lyophilized sponges loaded with (**c**) MIL-100(Fe), (**d**) ZIF-8, (**e**) bentonite, (**f**) celite 545, (**g**) ionene 3,3, and (**h**) ionene 6,6. The diffractogram of the base sponge (without fillers added) is shown in all cases for comparison.

Figure 3 shows the powder X-ray diffractograms (PXRDs) of chitosan powder, pectin powder, base sponges, and sponges with fillers, respectively.

Chitosan powder exhibits two characteristic peaks in the PXRD diffractogram at 2*θ* ≅ 10° and 20° (Figure 3a), which correspond to the crystallographic planes (020) and (110), respectively [84]. This semi-crystalline profile is associated with strong hydrogen bond interactions, favoring the attraction of polymer chains [85,86]. Pectin powder presents a series of very well-defined peaks on the PXRD diffractogram, indicating a crystalline structure (Figure 3b). Thus, the polymer powders of chitosan and pectin present a degree of crystalline structure, which remarkably can be observed in the case of pectin. However, it is worth noting that the base sponge presents a diffractogram associated with an amorphous material, without detecting the presence of peaks. Thus, the crystalline structure of the pectin is substantially reduced during the process of obtaining the sponges; hence, the crystalline pattern disappears or is completely masked by the amorphous one. This amorphous-like diffractogram was also obtained in the sponges loaded with fillers (Figure 3c–f), although very low-intensity peaks in the region of pectin diffraction were detected in those loaded with celite 545 and ZIF-8. Hence, it appears that most of the crystalline structures associated with the initial polymers are lost during the preparation of the sponge, resulting in a mainly amorphous final material with a low crystallinity degree.

The DSC plots of chitosan and pectin powders show endothermic peaks at 146 °C and 168 °C, respectively (Figure S3a,b, Supplementary Materials), which are associated with thermal disappearance of the crystalline phases at those temperatures, although with a low intensity (~1–5 Wg^−1^). In the case of the sponges, the thermal peaks appear in the intermediate region, between 146 and 168 °C (Figure S3c,d, Supplementary Materials). These results are consistent with the image of a mainly amorphous character of the sponge, although with the possibility of its forming localized ordered structures, contributing a very low degree of crystalline structure to the final material. The thermograms (Figure S4) show the characteristic range of biopolymer decomposition, which is usually associated with oligomerization of polymeric chains, observed here within the range of 125–225 °C, followed by graphitization at higher temperatures.

The properties of the sponges are directly associated with those of the precursor hydrogels. A notable central result is that the hydrogel obtained before the freeze-drying process is stable without observing phase separations, precipitations, or segregation of the fillers. This result is attributed to the incorporation of pectin, which provides a thickening effect and stabilizes the hydrogel. In fact, in the absence of pectin, neither of the two MOFs could be homogeneously dispersed in solutions of chitosan in acetic acid, and segregation was observed in many preparations. Additionally, pectin provides acidity to the medium, allowing the dissolution of chitosan without the need to dissolve the chitosan in acetic or lactic acid previously. In the present protocol, it is unnecessary to dissolve chitosan in a separate medium, which usually requires using acetic acid, but chitosan is added directly to the previously dispersed pectin solution. This represents an important improvement in comparison with a previously reported procedure [12] because it avoids introducing acetic acid to the formulation, which, due to its volatility, can expand inside the lyophilization equipment, with the possibility of damage (the vapor pressure at 25 °C is about 2 kPa for acetic acid, while it is only 0.001 kPa for lactic acid). Moreover, the present procedure is simpler, since all components are introduced in only one dissolution medium. Therefore, it is possible to obtain stable hydrogels of chitosan–pectin–MOFs without needing any additional acid (such as acetic acid, commonly used to dissolve chitosan). In summary, the use of pectin allows four important objectives to be reached: (i) to homogeneously and stably disperse the MOFs and clays in the hydrogel; (ii) to dissolve the chitosan without the need to incorporate acetic acid; (iii) to prepare the mixture of components in only one medium; and (iv) to obtain stable and soft composite sponges loaded with MOFs.

**Figure 4:**
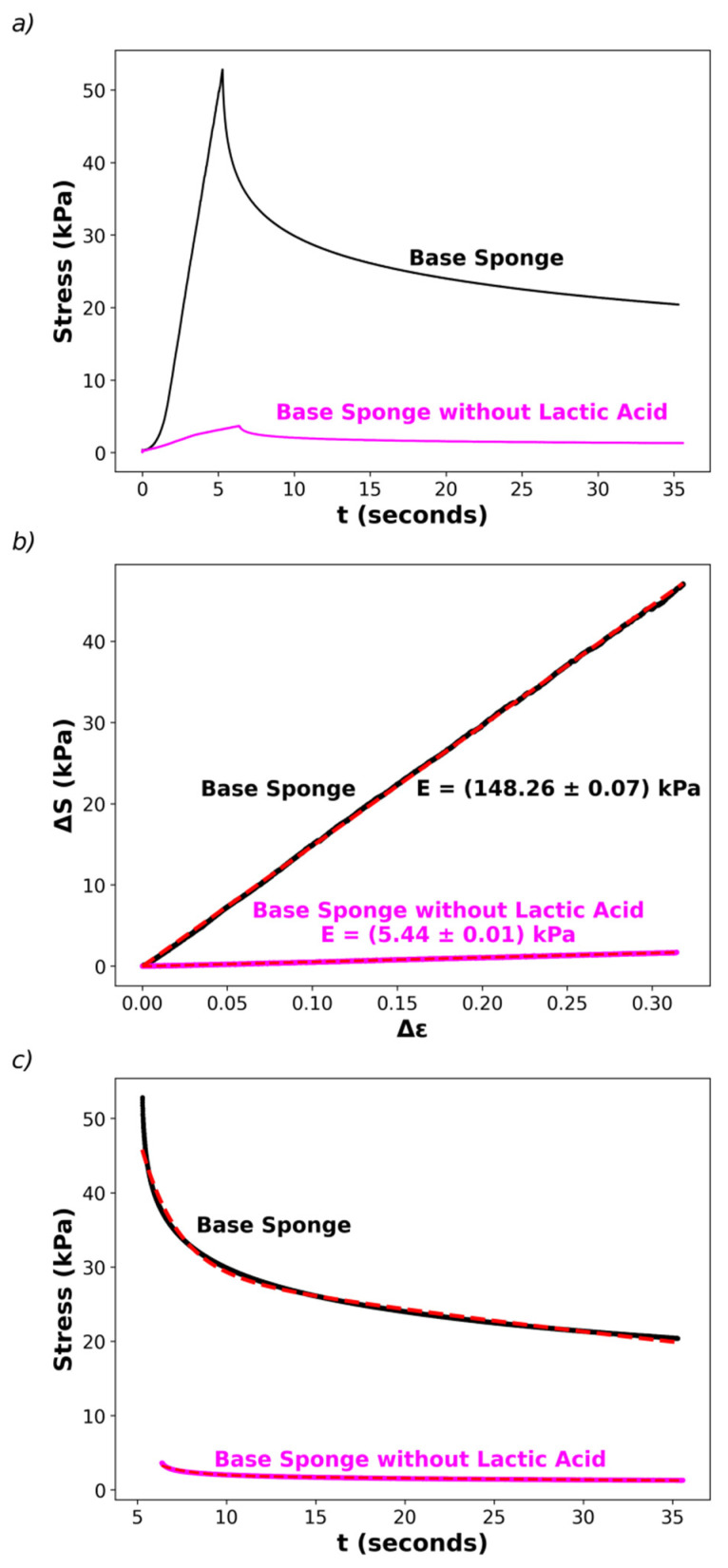
Compression tests for the base sponge and a sponge without lactic acid. (**a**) The stress (S) vs. time (t) during compression up to a strain (ε) of 40% (t_0_ ≅ 5–6 s); then the probe is released and the stress relaxation registered. (**b**) The change in stress, ΔS, as a function of the strain change, Δε. (**c**) Stress relaxation fitted by double exponential decays. The tests were performed at room temperature (22–25 °C).

Note that the lactic acid addition was not intended to lower the pH, which was already achieved by adding pectin without lactic acid, but to improve the mechanical properties of the final aerogel. Therefore, it is reasonable to assume that lactic acid acts as a plasticizer by modifying intermolecular interactions between chitosan or pectin chains. Preliminary studies concerning the elastic properties of the sponges were initiated in this work by determining stress–strain curves for the base sponge (containing lactic acid) and sponges without lactic acid (Figure 4) using a texturometer (as described in the Section 2).

Figure 4a shows plots of the applied stress, S, as a function of the compression time. The first part of the curves (at time t < t_0_, the peak of the stress in Figure 4a) was performed by compressing the sponges at a constant compression speed up to 40% strain. Then, the strain was held constant (t > t_0_, the second part of the curves in Figure 4a), registering the relaxation of the stress. The first part allows recovery of the Young’s modulus, E, by fitting the change in stress, ∆S, vs. the change in strain, ∆ε, as described in the Materials and Methods, observing that the experimental data follow Hooke’s Law, ∆S = E ∆ε, as shown in Figure 4b. The addition of lactic acid (25% weight percentage) in the hydrogels induces a huge increase in E, from E = (5.43 ± 0.01) kPa in the sponge without lactic acid to E = (148.3 ± 0.01) kPa (values corresponding to the recovered slopes of linear fits of ∆S vs. ∆ε). Thus, the sponges with lactic acid are more rigid, although they keep their elastic nature. This difference in behavior is also associated with the macroscopic behavior of the sponges when immersed in aqueous solutions: the sponges with lactic acid swell but do not disassemble, while those without lactic acid become disaggregated and finally transform into several small pieces dispersed in water.

Finally, it is interesting to note that stress time relaxations are similar for both sponges, with and without lactic acid. For instance, the second part of Figure 4a (t > t_0_) can be well-fitted by a double-exponential decay, S(t) = A_1_ exp(−(t − t_0_)/τ_1_) + A_2_ exp(−(t − t_0_)/τ_2_), where τ_1_ and τ_2_ are the relaxation times with relative weights A_1_ and A_2_, respectively. The fits are consistent with a short relaxation time in the order of τ_1_ ≅ (2.0 ± 0.1) s, with a relative weight of about 40% and a large relaxation time, τ_2_ ≅ (72 ± 4) s, contributing with a relative weight about 60% to the decay (fits shown in Figure 4c). These preliminary results suggest that the dynamic features associated with relaxation of the elastic parameters are similar in both sponges, even though there are differences in E. This observation warrants a deeper analysis of the elastic properties which is beyond the scope of the present work.

### 3.2. Protonation–Deprotonation Equilibrium in the Sponges

The chitosan–pectin interactions are discussed since they are relevant to the electrical charges in the sponge. The chitosan–pectin interaction is expected to be influenced by several factors, including (i) the electrical charges of the respective chains at the working pH (5.5 ± 0.5); (ii) the eventual binding between the -NH_2_ groups of the chitosan to carboxylate groups of pectin, thus crosslinking the chains of both polymers; and (iii) van der Waals interactions between both polymers, possibly enhanced by the presence of neutral sugar groups in the pectin. These factors are strongly influenced by the degree of methylation of the pectin, the number and chemical nature of neutral sugars in the branched lateral chains of the pectin, and the deacetylation percentage of chitosan. Thus, although the pectin–chitosan interaction in water dispersions has been considered attractive [87,88,89,90], an analysis of the mentioned factors is required and presented next.

**Figure 5:**
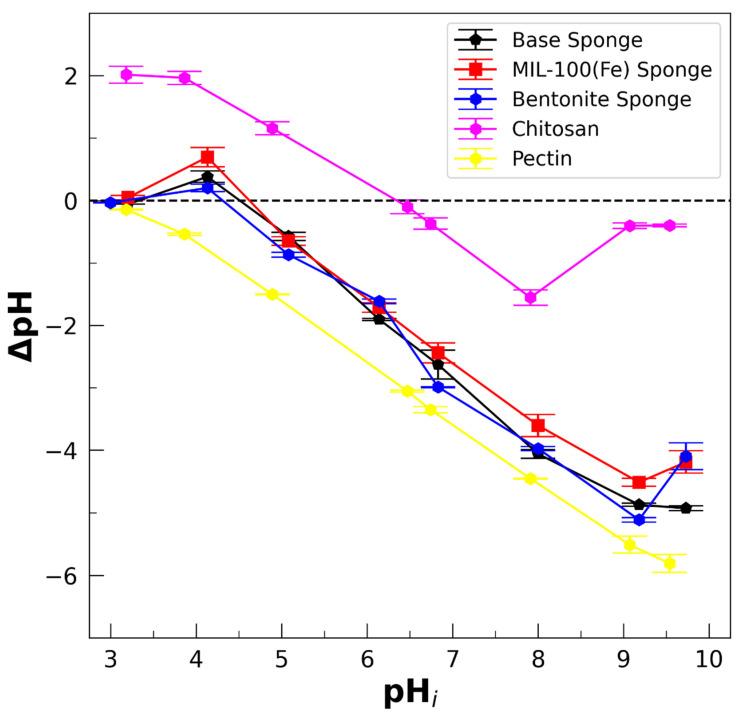
∆pH (= pH_f_ − pH_i_) vs. pH_i_ for determination of pH_PZNPC_ for chitosan powder, pectin powder, the base sponge, and sponges loaded with MIL-100(Fe) and bentonite. T = (296 ± 2) K; pH = (5.5 ± 0.5).

The ∆pH vs. pH result (Figure 5) is of the highest relevance for this analysis because ∆pH decreases with the difference between H^+^ and HO^−^, which can be connected to the charge balance in the considered material. For instance, the situation of ΔpH < 0 when a polymer or a material is introduced into an aqueous medium can be interpreted as an increase in the proton concentration in the solution because of the deprotonation of acid groups, indicating the presence and preponderance of anionic species in the polymer or material. Analogously, ΔpH > 0 indicates the protonation of basic groups and the prevalence of cationic species on the surface. The structures of chitosan, pectin, and lactic acid, showing the acid–base groups involved, are presented in Figure S2b,d (Supplementary Materials) in order to help with the following discussion.

First, aspects concerning the incorporation of pectin are discussed. The dominant structure of pectin is a chain formed by a linked (1 → 4) α-D-galacturonic acid backbone with a variable degree of methylation in the residues of the carboxylic acids. Native pectins are classified as highly methylated (HM) and lower-ester-content (LM) pectins. The backbone is interrupted by branched lateral chains rich in neutral sugars. For the pectin used here, the degree of methylation is between 60 and 70% (thus, it is considered as HM pectin), presenting ramifications of neutral sugars [80]. Hence, the acid–base properties of these types of pectin might be mainly determined by the carboxyl groups of the galacturonic acid and the degree of methylation [91] (pK_a_ of the galacturonic group = 3.5). In the case of LM pectins, pK_a_ = 2.9 was reported by Ralet et al. [91] and mentioned as close to 3.5 by Ström et al. [92]. For HM pectin, the pH of zero charge, pH_zcp_, determined by zeta-potential measurements, was estimated to be below 3 by Maciel et al. [93]. In agreement with this, the results of Figure 5 indicate that the point of zero net proton charge for our pectin powder is below 3. Therefore, the HM pectin used here must be considered as an anionic polymeric structure (referred to as pectate by Ralet et al. [91]) when dissolved at the pH of DI water (pH ≅ 5.5–6.0) in the first step of the preparation protocol (Figure S2a, Supplementary Materials) because of deprotonation of carboxyl acid groups.

Regarding chitosan, it is a linear polysaccharide composed of glucosamine units and *N*-acetyl-glucosamine units. Then, we determined by acid–base back-titration that the deacetylation degree of the chitosan is about 90%. It has been reported that the pK_a_ of chitosan with a high degree of deacetylation is between 6 and 7, in agreement with the pK_a_ of the glucosamine group. Additionally, the results of Figure 5 show that pH_PZNPC_ is around 6.5, with ∆pH > 0 for lower pHs. Thus, it is assumed that chitosan is positively charged at a pH < 6.5. Note that the magnitude of the absolute value of ∆pH is lower for chitosan than for pectin, suggesting there are fewer positive charges in chitosan than negative charges in pectin.

Concerning the lyophilized sponges, it must be noted that pectin is first dissolved in DI water (pH = 5.5–6.0) during the preparation protocol; thus, the solution reaches pH = 5.0 after dissolution, assigned to deprotonation of the carboxyl acid groups of the pectin. When lactic acid is added (pK_a_ = 3.9), the pH is further reduced to 4.5–4.0. Note that this pH is still very much above the pK_a_ and pH_PZNPC_ of the pectin; therefore, the pectin must be negatively charged due to deprotonated carboxylate groups (-COO^−^). Then, chitosan powder is added and the pH of the hydrogel solution is not altered, which is consistent with a lower number of protonated amino groups (-NH_3_^+^) in the chitosan than deprotonated carboxylic groups in the pectin, keeping between 4.5 and 4.0, below the pK_a_ and pH_PZNPC_ of chitosan (≅6.5). Thus, chitosan must be positively charged in the sponge, due to the presence of the -NH_3_^+^ group, although in a relatively low proportion in comparison to -COO^−^ in the pectin. The final pH of the solution is lower than the pK_a_ and pH_PZNPC_ of chitosan but higher than those of pectin, allowing the obtainment of polymers of opposite charges, favoring attractive interaction which contributes to stabilization, as observed. Therefore, the expected appropriate pH range for stabilizing the hydrogel is between 3.0 and 5.5, which is required to obtain polymers of opposite charges. It is worth noting that adding lactic acid at the used proportion preserves the pH within the mentioned limits. If the added amounts of lactic acid are too high and the pH is reduced below 3, then pectin will be in a neutral or cationic form by protonation of the carboxylic groups, which should destabilize the hydrogel. Thus, the added amount of lactic acid is a compromise between providing elasticity to the lyophilized sponge without destabilizing the previous hydrogel.

The pH_PZNPC_ of the lyophilized sponge was measured to determine the charge state of the sponge immersed in solutions. Figure 5 shows the curves of ΔpH vs. pH_i_, obtaining pH_PZNPC_ ≅ 4.5 for the lyophilized sponges. The point of zero proton charge, pH_PZNPC_, is assumed here as a good estimation of the zero-charge point, pH_zcp_, defined as the pH where the total charge on the sponge is zero [94]. The difference between pH_PZNPC_ and pH_zcp_ can be relevant in systems with high ionic strengths (>10^−1^ M), which do not obtain here. It was verified that this value is independent of the ionic strength of the solution, I, for I ≤ 10^−2^ M (by adding NaCl), and independent of the incorporated filler (Figure 5). Thus, it is reasonable to assume that pH_PZNPC_ ≅ pH_zcp_ for the sponges. Therefore, the structure of a lyophilized sponge is expected to have a negative electrical charge in the presence of DI water (pH = 5.5–6.0), which can be assigned to the deprotonation of the pectin backbone. As mentioned before, it was determined that pH_zcp_ ≅ 5.5 for chitosan; thus, the shift to pH_zcp_ ≅ pH_PZNPC_ ≅ 4.5 for the sponge is consistent with the presence of carboxyl acid groups when incorporating pectin. As we mentioned above, since the magnitude of the absolute value of ∆pH is lower for chitosan than for pectin, the number of positive NH_3_^+^ groups in chitosan is expected to be lower than the number of -COO^−^ groups in pectin, consistent with the lower pKa of -COO^−^ compared to the glucosamine group, yielding sponges characterized by the prevalence of carboxylates.

These results indicate that considering only point-charge electrostatic interactions, the sponges without fillers should exhibit preferential sorption of methylene blue (cationic, expected to interact with the -COO^−^ group) rather than methyl orange (anionic). However, this is not the case, as shown in the next sections. Therefore, other factors, such as van der Waals interactions and swelling of the sponge, must be considered when discussing the sorption of dyes.

### 3.3. Removal of Methyl Orange (MO) in Batches

**Figure 6:**
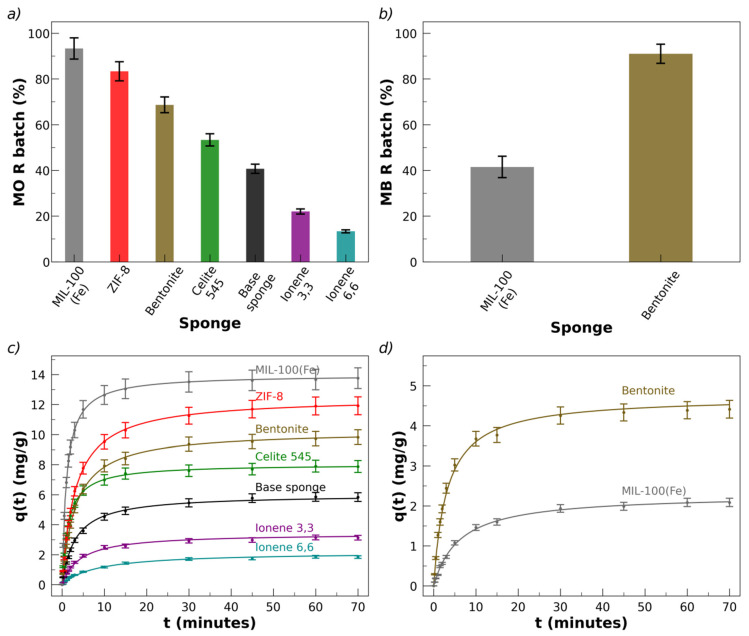
Batch removal of MO ((**a**,**c**), on the left) and MB ((**b**,**d**), on the right) for sponges loaded with different fillers. (**a**,**b**): Removal percentage, R, after reaching the sorption equilibrium. (**c**,**d**): q(*t*) as a function of time, *t*. *M_s_* = 30 mg, *V* = 10 mL, and C_0_ = 45 and 15 ppm for MO and MB, respectively. T = (296 ± 2) K; pH = (5.5 ± 0.5).

The percentage of MO removal, R, is presented in Figure 6a for the sponges with the different fillers.

Figure S5a (Supplementary Materials) shows pictures of methyl orange (MO) solutions (10 mL, 45 ppm) before and after being in contact (30 min, 130 rpm) with 30 mg of a blank sponge and a sponge with MIL-100(Fe) as filler. Figure S5c presents the visible absorption spectra of the remaining MO solution after contact with sponges containing different fillers, showing the decrease in light absorption relative to the original MO solution. These figures illustrate the color change of the solution after sorption of the dye into the sponges. An almost 100% removal of MO from the solution and its incorporation into the sponge was observed in the case of sponges with MIL-100(Fe). It was determined that, for a fixed dye concentration, the value of q*_e_* is independent of the mass of the sponge, *M_s_*, in the range of 10 mg ≤ *M_s_* ≤ 70 mg.

The order of increasing R according to the fillers is the following: MIL-100(Fe) > ZIF-8 > bentonite > celite > blank > ionene 3,3′ > ionene 6,6′. A base sponge only removes about 40% of MO; the removal percentage increases significantly when incorporating the fillers (except for the case of the ionenes, discussed separately). The larger values of R were obtained for the MOFs. MIL-100(Fe) is the best filler for sorbing MO into the sponges, with R > 90%. Since a negative zeta potential, ζ, has been reported for MIL-100(Fe) in aqueous solutions of pH > 4 [95,96], the localized charges on its crystals are expected to be negative. It is worth noting that the anionic dye is strongly removed by the anionic sponge, which has also incorporated a filler with negative surface charges. Figure 6c shows the results for MO kinetic removal in terms of q(t) vs. *t*.

The second-best absorber of MO is ZIF-8 (R ≅ 80%), which presents a positive surface charge independent of its crystal morphology, assigned to the exposed Zn^2+^ ions on the external surface [97,98,99,100]. The results indicate that the excellent sorption of the anionic dye MO by MIL-100(Fe) and ZIF-8 cannot be interpreted solely based on charge–charge electrostatic interactions, since both MOFs have electrical surface charges of opposite signs.

Following the decreasing order of sorbent fillers for MO are the clays (bentonite and celite 545). Bentonite colloids have an anionic character in the pH range considered here, due to the deprotonation of octahedral Al–OH and tetrahedral Si–OH groups and the partial replacement of cations in the internal structure by lower-charged cations [83]. Celite 545 is a diatomite earth whose surface silanol groups are deprotonated at pH < 8; thus, the surface charge of celite 545 and its zeta potential are negative in the considered pH range [101,102].

Concerning the dependence of q*_e_* on the dye concentration, C_o_, in the case of MO, the range of concentrations used was restricted to below 100 ppm (25 ppm ≤ **C_o_** ≤ 100 ppm), since several factors make both the tests and the interpretation of the results difficult at higher concentrations: dye aggregation is expected, the dye counter-ion concentration can influence the results, and the absorption of visible light at the peak of the spectrum is greater than 3, which makes spectrophotometric detection more complex. It was observed that **q*_e_*** varies linearly (and with zero ordinate) with **C_o_**, **q*_e_*** = K_H_ **C_0_**, for all the fillers considered, in the indicated concentration range (shown in Figure S5e,f, Supplementary Materials, for sponges with MIL-100(Fe)). The constant K_H_ is usually referred to as Henry’s constant, which can be interpreted as a magnitude proportional to the probability that a dye molecule is absorbed or adsorbed by a material. When considering the different fillers, the values of K_H_ followed the previously indicated order (MIL-100(Fe) > ZIF-8 > bentonite > celite > blank > ionene 3,3′ > ionene 6,6′). At a fixed **C_0_**, the parameter **q*_e_*** depends on the filler considered, with K_H_ = (0.290 ± 0.004) ppm^−1^mg g^−1^ for MIL-100(Fe). The values of K_H_ for the other fillers, relative to MIL-100(Fe), K_H_(filler)/K_H_(MIL-100(Fe)), are 0.85, 0.70, 0.55, 0.41, 0.22, and 0.12 for ZIF-8, bentonite, celite, the blank, ionene 3,3’, and ionene 6,6′, respectively. This indicates that, for example, the probability of a MO molecule being absorbed in a sponge with celite is 0.55 of the likelihood in a sponge with MIL-100(Fe), under the present conditions. The order of K_H_ with the fillers follows the same sequence as **R**, shown in Figure 6a. All tests were replicated three times with newly prepared sponges, obtaining very low dispersions of the equilibrium and kinetic parameters.

The values of q_e_ vs. C_e_ (the equilibrium concentration of dye in the solution after sorption) were well fitted by the Langmuir equilibrium model, q_e_ = *q_max_* C_e_/(1 + *K_L_*C_e_) (shown in Figure S5f, Supplementary Materials, for the cases of fillers with maximum removal), where *q_max_* is the maximum value of q_e_ and *K_L_* is the Langmuir equilibrium constant. For instance, the fits are consistent with *q_max_* = 53 mg/g and *K_L_* = 0.15 L mg^−1^ (≅0.01) for MO in sponges loaded with MIL-100(Fe). Values of *q_max_* of about 25–40 mg/g, similar to those obtained here, were recently reported by Kloster et al. [64,66] for the absorption of red congo (anionic dye) in systems based on chitosan cross-linked with glutaraldehyde and modified cellulose aerogels.

The reuse of the sponges was tested in five cycles of sorption–desorption. For desorption, the sponges loaded with MO were immersed in clean solutions of pH = 4.0 (HCl). The release of the dye into the solution was observed immediately afterwards. The release was complete in all the sponges in about 60 min. The sponges were preserved after desorption. Then, the clean sponges were reused in another MO sorption–desorption cycle, as illustrated in Figure S6 (Supplementary Materials), with a decrease in R % from (93 ± 5)% to (84 ± 3)% after five cycles.

### 3.4. Removal of Methylene Blue (MB) in Batches

Figure S5b (Supplementary Materials) shows pictures of MB solutions before/after contact with sponges loaded with bentonite and MIL-100(Fe), where the color change can be observed due to sorption of the dye into the sponges. Figure S5d (Supplementary Materials) presents the respective visible absorption spectra of the solutions, showing the decrease in the spectra after sorption. The removal percentage of MB is about 40% and 90% for sponges loaded with MIL-100(Fe) and bentonite, respectively, as shown in Figure 6b. All removal tests were replicated three times with newly prepared sponges, obtaining very low dispersions of the equilibrium and kinetic parameters. The error bars in Figure 6b are standard deviations of the results of three replicated essays. The kinetic plots, q(*t*) vs. *t*, for all sponges, averaging the results of the three repeats, are presented in Figure 6d, showing the standard deviations of the replicates.

Surprisingly, MB is not sorpted by the base sponge and is only sorpted by two of the six sponges with fillers. Under the conditions essayed here, the only systems that remove MB are the sponges containing bentonite and MIL-100(Fe). Another difference in comparison with MO is that the order of sorption is altered: sorption of MB is higher in sponges loaded with bentonite than in those with MIL-100(Fe) (Figure 6b,c). Furthermore, the Henry constant, K_H_, for MB is much lower than for MO in sponges containing bentonite and MIL-100(Fe): K_H_ = (0.09 ± 0.01) ppm^−1^mg g^−1^ for sponges with bentonite and K_H_ = (0.04 ± 0.01) ppm^−1^mg g^−1^ in sponges with MIL-100(Fe), which values are more than three times lower than the values for MO. A possible hypothesis to rationalize the null removal of MB in most sponges and the relatively low K_H_ values of those with bentonite and MIL-100(Fe) could refer to the electrical charges of the sponges and MB. The base sponge and MB had the same charge sign at the pH and ionic strength of the removal experiments, which could have contributed to rejection of the MB. The incorporation of bentonite or MIL-100(Fe) into the sponges, both anionic fillers, may favor the sorption. However, MB is not sorpted by sponges loaded with the anionic celite 545. This suggests that the addition of negative charges by celite 545 is not effective enough and/or that other factors must influence the rejection, such as specific van der Waals interactions between MB and the components of the sponge. The equilibrium Langmuir isotherms are consistent with *q_max_* = 21 mg/g and *K_L_* = 0.20 L mg^−1^ for MB in sponges loaded with bentonite.

As mentioned in the case of MO, other factors beyond the net charge–charge attraction of repulsion must be considered in the sorption of the dyes. In particular, van der Waal interactions between dye and MOFs, π-π stacking with the phenyl moiety in the case of MIL-100(Fe), and incorporation of the dye into the pores of the MOFs should play a predominant role. These factors, which are usually invoked to justify the excellent adsorption of target compounds on MOFs, seem to apply effectively in the case of MO since the better absorbers are two MOFs of opposite surface electrical charges. In addition, the electron density on the highest occupied molecular orbital (HOMO) of the dyes must be considered. In the case of MB, the electron density on the HOMO is very homogeneously distributed along the very symmetric molecular structure, as reported by Ullah et al. [103], using DFT calculations. In the case of MO, the presence of the -SO_3_ terminal group at one extreme of the molecular structure induces spatial asymmetries in the charge distribution, with a higher electron density on the -SO_3_ moiety and a lower density on the other, as calculated by DFT, informed by You et al. [104] for the non-protonated MO (note that pKa varies between 3.0 and 4.0, as has been reported for MB [105]; thus, the central azo group is not protonated at the working pH, 5.0–6.0). Therefore, the asymmetric electron density of MO on its HOMO suggests the possibility, to be explored in further works, of more intense and directional electrostatic interactions of MO than MB with functional groups of the chitosan–pectin structure, which is in agreement with the selective sorption between both dyes observed in the present work.

### 3.5. Analysis of the Kinetic Removal of MO and MB

**Figure 7:**
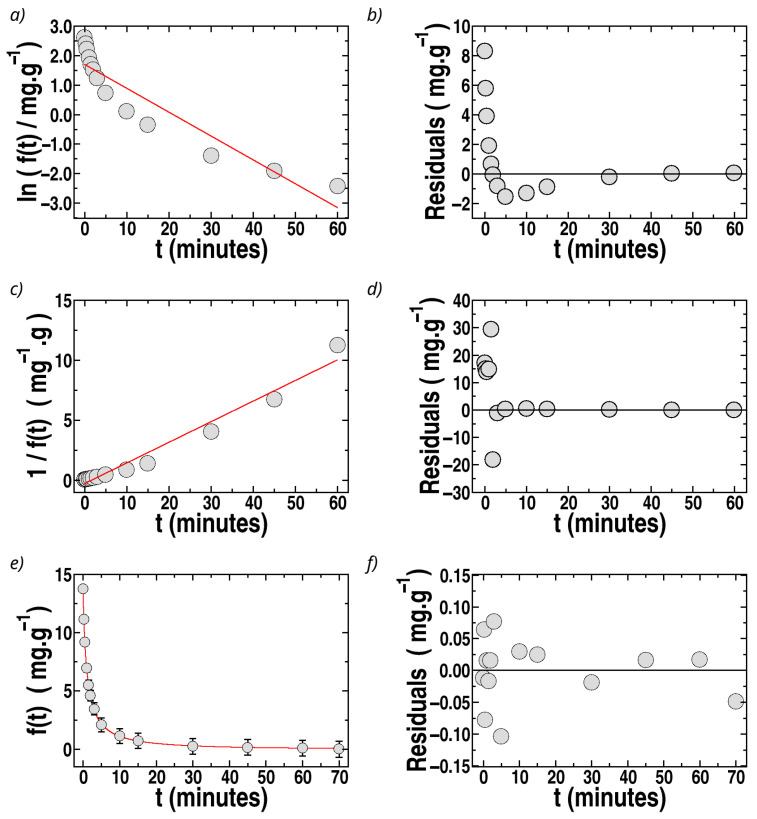
Fits of the kinetic data for MO sorption in sponges with MIL-100(Fe). The circles indicate experimental data. The solid (red) lines are the results of fits: (**a**) Ln *f*(*t*) vs. *t* (first-order). (**c**) 1/*f*(*t*) vs. t (second-order). (**e**) *f*(*t*) vs. *t* fitted by Equation (10). (**b**,**d**,**f**): Residuals for each model, calculated as the difference between the experimental and recovered values of *f*(*t*). *M_s_* = 30 mg, **C_o_** = 45 ppm, *V* = 10 mL. T = (296 ± 2) K; pH = (5.5 ± 0.5).

The experimental kinetic sorption curves of **q**(*t*) as a function of time, *t*, for all the fillers, are illustrated in Figure 6c (MO) and Figure 6d (MB). The features of the sorption kinetics are discussed in this section. For the simplicity of the mathematical treatment, the function, *f*(*t*), defined as

(1) $$f\left(t\right)=q_{e}-q(t)$$

was used to describe the experimental kinetic data of sorption into the sponges as a function of time, *t*, with the constraints $f\left(t=0\right)=q_{e}$ and $f\left(t\to \infty \right)$ = 0, expressing *f*(*t*) in mg g^−1^. The experimental results of *f*(*t*) vs. *t* for sponges with MIL-100(Fe), the filler with the highest sorption for MO, are shown in Figure 7, and similar features were observed for sponges loaded with all the fillers.

The fit of *f*(*t*) by first-order kinetics is illustrated in Figure 7a for the case of MO sorpted by sponges containing MIL-100(Fe). It can be observed that a straight line cannot fit Ln *f*(*t*) vs. *t*. Furthermore, the residuals (differences at each time, *t*, between the experimental data and the fit) are strongly time-dependent (Figure 7b). Thus, the experimental data cannot be described by a first-order model. Similar results were observed for all sponges loaded with different fillers.

**Figure 8:**
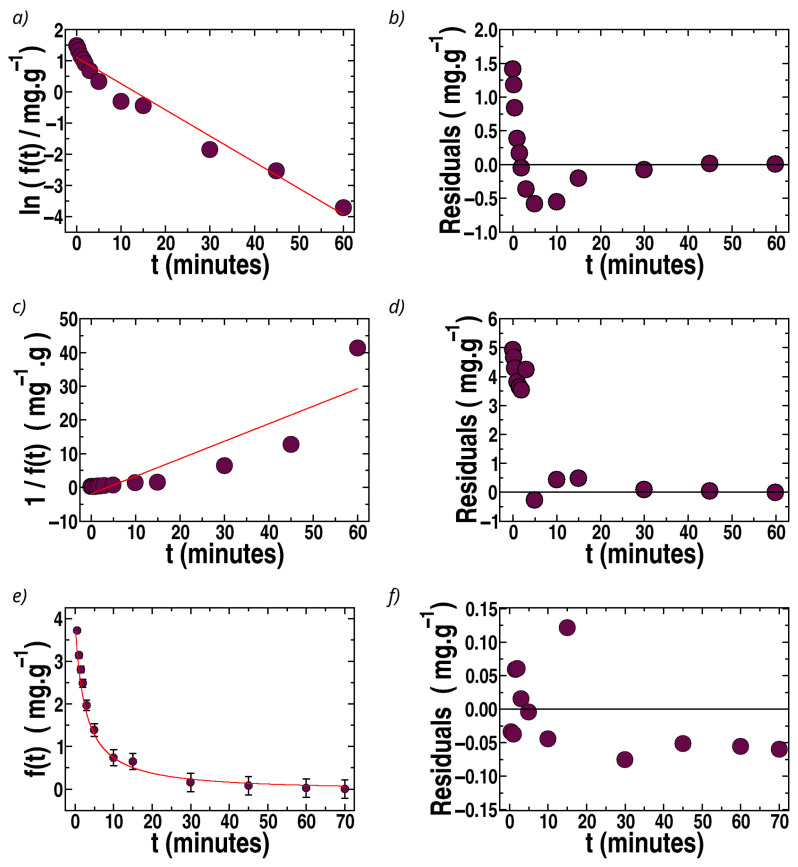
Kinetic data for MB sorption (batch) in sponges with bentonite. Circles: experimental data. Solid (red) lines: Tesults of fits. (**a**) Ln *f*(*t*) vs. *t* (first order). (**c**) 1/*f*(*t*) vs. t (second order). (**e**) *f*(*t*) vs. *t* fitted by Equation (10). (**b**,**d**,**f**): Respective residuals (differences between experimentally recovered values of *f*(*t*)). *M_s_* = 30 mg, **C_o_** = 15 ppm, *V* = 10 mL. T = (296 ± 2) K; pH = (5.5 ± 0.5).

The fits by second-order kinetics:

(2) $$f\left(t\right)=\frac{q_{e}}{1+{k_{2nd}q}_{e}t}$$

where $k_{2nd}$ is the second-order rate constant, are better than those by first-order kinetics but still unsatisfactory, with time-dependent residuals, as shown in Figure 7c,d for the case of sponges loaded with MIL-100(Fe). It is important to remark that it is frequently affirmed in the literature that adsorption or absorption data follow second-order kinetics based solely on the argument that plots of *t*/*q*(*t*) vs. *t* (or 1/*f*(*t*) vs. *t*) are well-described by a straight line, as predicted by second-order equations. However, this criterion must be supported additionally by the analysis of the residuals, verifying whether they follow a random dependence on the independent variable, *t*, and by analyzing how close to unity are the values of *R*^2^. In the present case, the fits by second- and first-order kinetics do not satisfy the criterion of time-independent residuals, as can be observed in Figure 7c,d and Figure 8c,d, and the values of *R*^2^ are not acceptable in most of the cases, as shown in Table 1.

Therefore, the kinetics of the process is neither first- nor second-order, which motivated the search for an appropriate model. Hence, we explored ideas taken from so-called Langmuir kinetics, described by Liu and Shen [106] and Azizian [107] for the case of surface adsorption. In Langmuir kinetics, which must not be confused with the Langmuir equilibrium isotherm of **q*_e_*** vs. **C_e_**, both the adsorption and desorption processes are incorporated as simultaneously occurring.

Since the early work of Langmuir, the adsorption–desorption of analytes has been considered the basis for understanding different surface processes, including the removal of dyes by solid surfaces. Consequently, Liu and Shen [106] and Azizian [107], following Langmuir, proposed a mathematical model that includes adsorption and desorption to and from a generic surface to describe the removal kinetics in specific cases. This model implies the presence of adsorption sites on the adsorbent surface, where the adsorbate is attached. Therefore, all the terminology and concepts presented by Liu and Shen and Azizian are those associated with surface phenomena, i.e., with adsorption processes. However, the mathematical treatment can be extended to situations where there are (i) a finite number of reception sites for a solute (bound state) and (ii) kinetic processes for the interconversion of the solute between the bound and unbound states. This situation can arise, for example, in the case of biopolymeric matrices with a significant capacity for swelling of their structure upon contact with an aqueous medium, allowing the solute to enter their interior. Therefore, swelling allows the dye not only to interact with the surface of the matrix but also to incorporate itself into the material by accompanying the solvent in the swelling process. In this way, we hypothesize in the next paragraphs that swelling allows the solute to access incorporation sites, which could be in its internal core, not only in the most external regions. It should be noted that swelling connects internal areas of the material with external ones. Thus, the interior of the material, through the intermediary action of the swelling process, is considered a region with binding sites, which have been brought into contact with the aqueous solution by the swelling process. If we also consider a finite number of internal binding sites, then the entire process can be treated mathematically in the same way as Liu and Shen did for surface processes. In this case, it is not appropriate to refer to the processes as adsorption, since the phenomenon does not occur on the external surface but rather throughout the whole matrix, but it may also be questionable to refer to absorption, since the solution penetrates the entire material. Therefore, using the term sorption, we demonstrate below that the ideas from Liu and Shen, as well as Azizian, describe the observed kinetics satisfactorily. It is proposed here that the lyophilized sponge presents sites “B” for the sorption and desorption of the dye, according to the following scheme:

(3) $$k_{s}\;A+B⇄AB\;k_{d}$$

where A and AB represent the target dye and the sorption complex, respectively. The constants $k_{s}$ and $k_{d}$ indicate the sorption and desorption kinetic constants. The time derivative of the fraction of occupied sites in the sponge at time *t*, referred to as *θ*(*t*), is given by

(4) $$\frac{d\theta }{dt}=k_{s}\left[A\right]\left(t\right)\left(1-\theta \right)-k_{d}\theta$$

with

(5) $$\theta \left(t\right)=\frac{q(t)}{q_{max}}$$

and

(6) $$\left[A\right]\left(t\right)={\left[A\right]}_{0}-q_{max}X$$

where *q_max_* is the maximum value of **q**, which corresponds to *θ* = 1 (*q_max_* and **q*_e_*** expressed in mg/g), $\left[A\right]\left(t\right)$ is the molar concentration (expressed in M = mol L^−1^) of the dye in the solution at time *t*, ${\left[A\right]}_{0}$ is the initial molar concentration, and *X* is defined as

(7) $$X=\frac{M_{s}}{{VM}_{r}}$$

where *M_s_* is the mass of the sponge (g), *V* is the volume of the solution (L), and *M_r_* is the molar mass of the dye (327.33 and 319.85 g mol^−1^ for MO and MB, respectively). Note that [*A*]_0_ (M) = **C_0_** (ppm)/1000 *M_r_* (gmol^−1^), which is in the order of 10^−4^ M under the present conditions.

It can be demonstrated by combining Equations (1)–(7) that the function *f*(*t*), defined in Equation (1), should satisfy the following differential equation:

(8a) $$\frac{-df}{dt}=k_{1}f\left(t\right)+k_{2}{f(t)}^{2}$$

(8b) $$\frac{d\theta }{dt}=k_{1}(\theta_{e}-\theta )+{k_{2}q}_{max}{\left(\theta_{e}-\theta \right)}^{2}$$

where $k_{1}$ and $k_{2}$ are fitting parameters. The units of $k_{1}$ are min^−1^, while $k_{2}$ is expressed in units of mg^−1^g min^−1^. The fitting parameters $k_{1}$ and $k_{2}$ are related to $k_{s}$, $k_{d}$, and ${\left[A\right]}_{0}$ via

(9a) $${\left(k_{1}\right)}^{2}={\left(k_{s}\right)}^{2}{\left({\left[A\right]}_{0}-q_{max}X\right)}^{2}+{2k}_{s}k_{d}\left({\left[A\right]}_{0}+q_{max}X\right){+(k_{d})}^{2}$$

(9b) $$k_{s}=\frac{k_{2}}{X}$$

Note that $k_{1}$ (Equation (9a)) is an effective constant that contains $k_{d}$, $k_{s}$, and ${\left[A\right]}_{0}$, representing the complexities involved in the sorption and desorption processes. The constants $k_{2}$ and $k_{s}$ are only associated with the sorption, not the desorption, and are not dependent on ${\left[A\right]}_{0}$. The analytical solutions of Equation (8), under the conditions $f\left(t=0\right)=q_{e}$ and $f\left(t\to \infty \right)\;$= 0 are

(10) $$f\left(t\right)=\frac{(k_{1}/k_{2})}{\left(\left(1+\left(\frac{k_{1}}{q_{e}k_{2}}\right)\right)exp\left(k_{1}t\right)-1\right)}\;\;\;if\;k_{1}\neq 0\;(Langmuir)$$

Equation (10) is only valid if $k_{1}\neq 0,$ otherwise the expression for second-order kinetic, recovered from Equation (8) for $k_{1}=0,$ must be considered.

The experimental data for *f*(*t*) vs. *t* were fitted by Equation (10), obtaining excellent fits for all sponges with the different fillers. The results of the fits and the residuals are plotted as a function of time in Figure 7e,f for the case of MO removal by sponges loaded with MIL-100(Fe) (the best filler for MO sorption). The residuals are about ten times lower than those for second-order fits, and no tendency of the residuals with *t* can be observed. The recovered values of $k_{1}$, $k_{2}$, and *R*^2^ for MO in all sponges are presented in Table 1, observing excellent values of *R*^2^ with a remarkable improvement compared to fits by second-order kinetics. The sorption constant, *k*_*s*_, was also calculated using Equation (9b) (Table 1). The percentage errors are 10–20% for $k_{1}$ and 15–40% for $k_{2}$ and $k_{s}$. In the case of ionenes, the fits provide the lowest *R*^2^ and the highest error for the recovered constants (80% error of $k_{s}$ for ionene 3,3′), associated with the destabilizing effect of ionenes on the sponge structure. Among the other fillers, MIL-100(Fe) and celite 545 present the largest $k_{s}$ values.

The fits of *f*(*t*) vs. *t* by first-order, second-order, and Langmuir kinetics for the case of MB are illustrated in Figure 8 for sponges loaded with bentonite (the best filler for MB).

**Figure 9:**
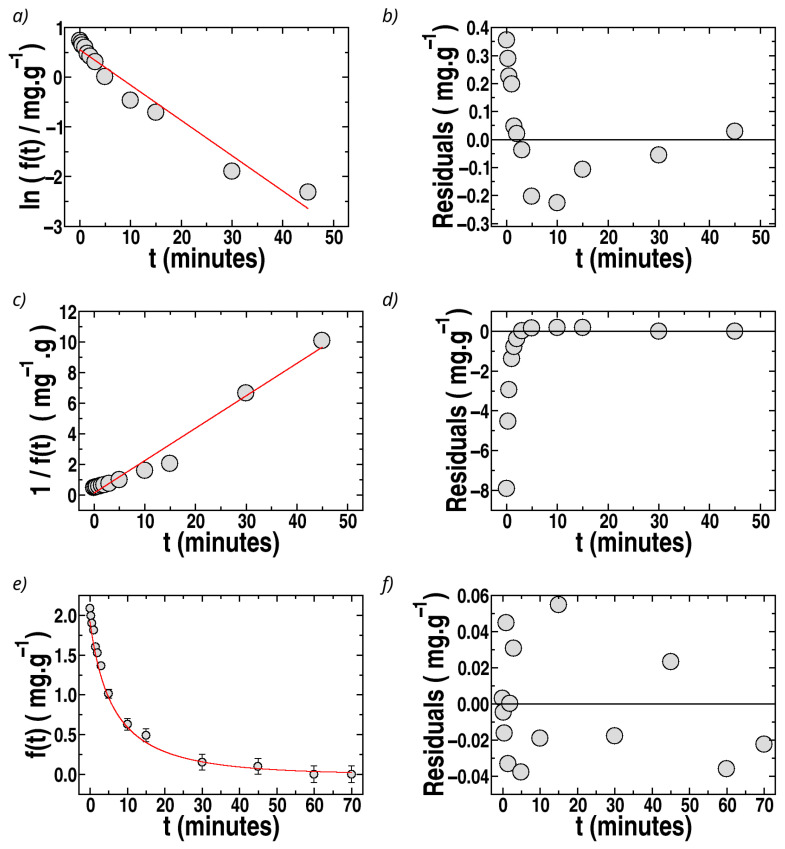
Fits of the kinetic data for MB sorption (batch) in sponges with MIL-100(Fe). Circles: experimental data. Solid (red) lines: fits. (**a**) Ln *f*(*t*) vs. *t* (first-order). (**c**) 1/*f*(*t*) vs. t (second-order). (**e**) *f*(*t*) vs. *t* fitted by Equation (10). (**b**,**d**,**f**): Respective residuals (differences between experimentally recovered values of *f*(*t*)). *M_s_* = 30 mg, **C_o_** = 15 ppm, *V* = 10 mL. T = (296 ± 2) K; pH = (5.5 ± 0.5).

The case of MB removal by sponges with MIL-100(Fe) is shown in Figure 9. The correlation coefficients and recovered parameters, using Langmuir kinetics, are shown in Table 2 for sponges loaded with bentonite and MIL-100(Fe).

Recovery of the desorption rate constant, $k_{d}$, from Equation (9a) is indirect and not straightforward since the uncertainties of the fits influence it, propagated through the second-grade polynomial equation. However, an analysis of consistency using the recovered values of *k*_1_ and *k*_2_ indicates that positive values of *k_d_* from Equation (9a) can be estimated only when considering relatively low values of *q_max_* (*q_max_* < 100 mg/g in all cases). This agrees with the *q_max_* values indicated in the previous sections; *q_max_* = 53 mg/g for MO in sponges with MIL-100(Fe) and 21 mg/g for MB in sponges loaded with bentonite. Additionally, the value of *k_d_* can be estimated using *k_s_* and *K_L_* in the mentioned cases of maximum removal (sponges loaded with MIL-100(Fe) for MO and loaded with bentonite for MB) via

(11) $$K_{L}=\frac{\theta_{e}}{C_{e}(1-\theta_{e})}=\frac{k_{s}}{k_{d}}$$

with *K_L_* = 0.15 Lmg^−1^ (4.9 × 10^4^ M^−1^) for MO in sponges loaded with MIL-100(Fe) and *K_L_* = 0.20 Lmg^−1^ (6.4 × 10^4^ M^−1^) for MB in sponges loaded with bentonite, with the values of *k_s_* reported in Table 1 and Table 2. Thus, *k_d_ ~* 0.16 min^−1^ and 0.17 min^−1^ are estimated for MO (in sponges with MIL-100(Fe)) and MB (in sponges with bentonite), respectively.

The rate constants recovered by the Langmuir kinetics of Table 1 and Table 2 (MO and MB, respectively) can be compared. As mentioned above, $k_{1}$ is an effective constant that contains $k_{d}$, $k_{s}$, and **C_0_**. The constant $k_{2}$ is only associated with the sorption, not desorption, and is not dependent on **C_o_**. In the case of sponges loaded with MIL-100(Fe), the values of $k_{1}$, $k_{2}$, and $k_{s}$, are very similar for MO and MB, but lower values of *k_d_* are estimated for MB. For sponges loaded with bentonites, $k_{2}$ and $k_{s}$ are slightly larger for MB than MO, which could be associated with the anionic and cationic characters of bentonite and MB, respectively.

The comparison of the recovered constants with values reported in the literature is not straightforward for different reasons. The first difficulty is that we could not find articles where the kinetic data for dye removal in chitosan or pectin were adjusted using Langmuir kinetics. In fact, in all the articles found for the removal of MO and MB in chitosan hydrogels or membranes, the data were adjusted using second-order kinetics, reporting $k_{2nd}$ values [30,31,32,33,35]. However, our data are not adjusted by second-order kinetics, making the comparison meaningless. In both cases, MO and MB (Table 1 and Table 2), the only acceptable fit, according to the visual observations and the analysis of the determination coefficient, *R*^2^, and considering the residuals, is provided by Langmuir kinetics. Table 3 presents the values of *R*^2^ obtained for the different fits (first-order, second-order, and Langmuir kinetics), where it can be observed that a large improvement was obtained in the case of Langmuir kinetics for all the sponges (changing the filler).

Langmuir kinetics is the only one of the considered models that provides excellent fits and a rational basis for understanding the kinetic behavior. Although the pseudo-second-order fits can be used as a coarse approximation, Langmuir kinetics is the only model that is statistically acceptable in the present case and which provides expressions for the constants *k*_s_ and *k_d_* based on sorption and desorption competing processes and not as an empirical or practical approximation. Nevertheless, based on the recovered *k*_1_ and *k*_2_, it can be understood why the empirical fits by pseudo-second-order kinetics are currently presented in the literature on related systems. For that, it must be noted that Langmuir kinetics can be interpreted as a mixture of first- and second-order kinetics, whose percentage contributions can be obtained when analyzing Equation (8b), which provides (d*θ*/dt). For instance, the percentage contribution of first orders to d*θ*/dt is equal to 100 *k*_1_/(*k*_1_ + *q*_max_*k*_2_), which is about 1% in our cases. Therefore, comparing *k*_2_ from Table 1 and Table 2 with the *k*_2*nd*_ values reported for similar systems seems reasonable, since they have the same units. Table 4 presents a comparison of the sorption parameters of MB and MO with those previously obtained by other authors for related biopolymers, showing that those reported here have a similar order of magnitude. Values of $k_{2nd}$ of the order of 10^−2^ g mg^−1^ min^−1^ have been reported by Bahrudin et al. [30] and Zeng et al. [32] for MO and by Fan et al. [35] and Lee et al. [70] for MB, which are of the same order of magnitude as those for the $k_{2}$ constants presented in Table 1 and Table 2. Thus, the chitosan–pectin–lactic acid aerogels exhibit high removal % values at environmentally relevant dye concentrations, especially in the case of MIL-100(Fe) for MO, and, in the case of MB, they show a significant increase in removal when incorporating MIL-100(Fe). The kinetic and equilibrium parameters at room temperature and pH 5.5 are similar to many of those previously reported in the literature for chitosan matrices with other fillers. Additionally, in comparison with the absorption of red congo in cross-linked chitosan aerogels reported by Kloster et al. [64], the kinetics presented here are one order of magnitude faster.

### 3.6. Effect of Ionenes and Influence of Ionic Strength (I)

The case of ionenes as fillers requires special attention since they are polymeric chains with net positive charges on their structures, compensated by the negative counter-ions (bromide in the present case). Therefore, it could be thought that there is a strong affinity between the structures of the sponge and the ionenes since they present net electrical charges of opposite signs. In particular, important stabilization effects could be imagined between the chains of ionenes and pectin through attractive electrostatic interactions. Although those effects are present, they seem not strong enough to generate stable sponges (loaded with ionenes) in contact with aqueous solutions of low ionic strength. On the contrary, these systems are the only unstable ones, since these sponges gradually disassemble after three hours of contact with the external solution. Several factors must be considered to analyze this effect, such as the charge and nature of the bromide counter-ion, the interactions between ionene chains, and the uptake of water by them [73]. Based on our observation of the sponges loaded with ionenes when immersed in aqueous solutions of low ionic strength, water uptake appears as a central factor. When a portion of the sponge loaded with ionenes is placed in contact with a low-ionic strength solution, high swelling of the sponge is observed, much greater than that observed for the base sponge and sponges loaded with other fillers. Water produces a high degree of swelling, which continues and induces, after 3 h, the gradual dismemberment of the sponge. The process ends with its total fractionation into multiple small portions after 5 h from immersion. Consequently, the instability of these sponges seems to be a consequence of the high degree of swelling. Motivated by these observations, an attempt was made to reduce the degree of swelling by increasing the ionic strength of the aqueous solution (I ≅ 1 M, adding NaCl, KNO_3_, or NaI). An increase in osmotic pressure is expected in the opposite direction to the entry of water into the sponge because the water molecules should be maintained by hydrating the ions added to the outer side of the sponge. In agreement with this hypothesis, we have observed that the swelling degree decreases noticeably with increasing ionic strength in all sponges, particularly in those loaded with ionenes. These sponges are now very stable in the medium with a high ionic strength, with no dismemberment observed for at least one week. All sponges in high-ionic strength medium remove MO, and those with bentonite and MIL-100(Fe) continue to remove MB. However, we have observed in preliminary tests that the removal rate decreases noticeably with increasing ionic strength and that the incorporation of dyes begins at the surface of the sponge, where pre-concentration of the dyes was observed. These observations are consistent with a lower and slower incorporation of water and dyes into the sponge. On the one hand, swelling at high ionic strength stabilizes all sponges, particularly those loaded with ionenes, such that their disintegration is avoided. On the other hand, it slows down the kinetics of absorption of dyes, such that, for example, the total removal of MB from sponges with bentonite in times of 24 h for ionic strength of the order of 1 M was observed. Thus, the present results indicate that a systematic analysis of the effect of ionic strength on sponge stability, dye removal efficiency, and kinetic factors appears to be of enormous importance, although outside the scope of the present work.

### 3.7. Preliminary Flow Tests with MO and MB

**Figure 10:**
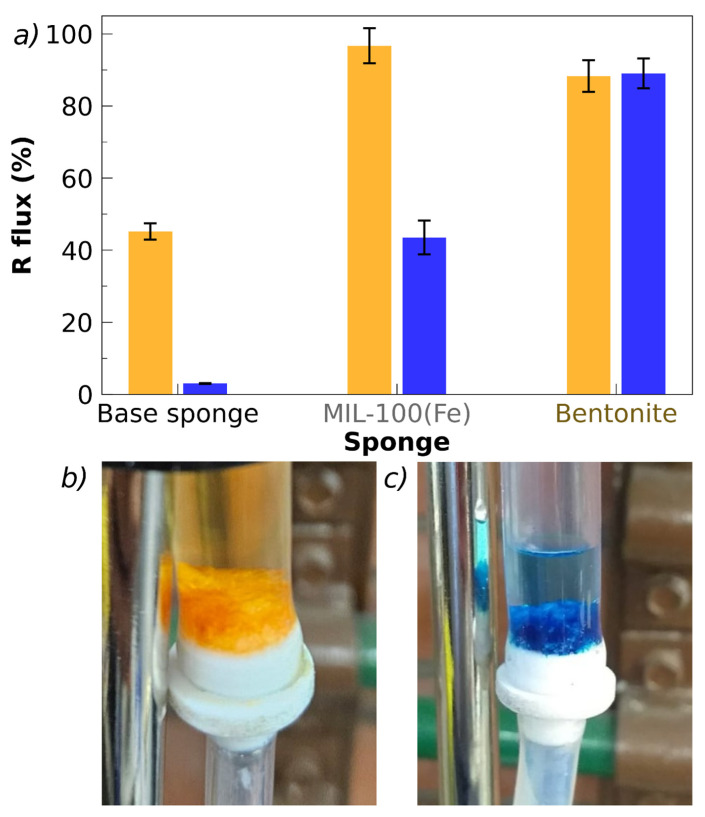
Flow removal tests. (**a**) Removal percentage (R) for MO (orange bars) and MB (blue bars), removed by the base sponge, sponges with MIL-100(Fe), and bentonite. (**b**,**c**) Sponges in the flow set-up after passing a flow of MO ((**b**) filler: MIL-100(Fe)) and MB ((**c**) filler: bentonite), from above. Conditions: flux: 2.5 mL/min; flowing time = 25 min (discarding the first 5 min). Retention time ≅ 0.5 min, *M_s_* = 30 mg, **C_o_** = 45 ppm (MO), and 15 ppm (MB). T = (296 ± 2) K; pH = (5.5 ± 0.5).

The presented flow tests should be considered only as a preliminary assessment of the subject of flow testing, carried out to explore the capacity of the sponges. These issues require specific and exhaustive studies that are beyond the scope of this work. In particular, the comparison of batch and flow results should be considered exclusively as an indication of the material’s potential, given the relatively low statistical volume obtained at present and the fact that the flow test conditions must be optimized. A central parameter, whose influence on flow removal must be studied in detail, is the retention time, *t_r_*, which can be defined as the ratio between the sponge volume (*V_sponge_*) and the applied flow, *F*: *t_r_* = *V_sponge_*/*F*. In the tests performed, *V_sponge_* = 1.3 cm^3^ and *F* = 2.5 cm^3^/min, and therefore *t_r_* = 0.5 min. Considering that the total experimental time was between 20 and 30 min, approximately 40 to 60 volumes of solution were passed through the sponge during one test. These conditions are expected to have influenced the removal percentages achieved. For the tests reported here, conducted under these conditions, the results are presented in Figure 10. The removal tests in flow were performed for MO and MB using sponges loaded with MIL-100(Fe) (for MO) and bentonite (for MB).

Sponges with a total mass, *M_s_* = 30 mg were used, with the same weight percentages of chitosan, pectin, lactic acid, and fillers as in the batch experiments. The initial (injected) dye concentration was 45 and 15 ppm for MO and MB, respectively, the same as in the batch experiments. In the case of MO, the base sponge achieves a 40% removal under flow conditions, while the presence of bentonite and MIL-100(Fe) increases the removal up to 85 and 95%, respectively. In the case of MB, the flow removal by the base sponge is below 2% and the addition of MIL-100(Fe) only increases R to 40%, while the highest removal percentage is obtained with bentonite (85%) (Figure 10a,b). Under the applied conditions of flow, retention time, and total time of the tests, the removal results are quite similar to those observed under batch conditions, suggesting that saturation of the removal capacity of the sponges may be reached. Pictures of the sponges after passing through the dye solutions are shown in Figure 10b,c observing the color associated with the incorporation of the dyes and the difference in color between the injected and filtered solutions. These preliminary results under flow conditions are auspicious since they show that the freeze-dried sponges do not obstruct the passage of the solution to be remediated under the conditions tested.

## 4. Conclusions

The implemented preparation protocol of freeze-dried sponges constitutes a relevant improvement concerning the loading of MOFs. It allows the complete incorporation of MOFs with a homogeneous distribution of fillers and avoids the use of acetic acid. The incorporation of pectin plays a central role, providing two simultaneous effects: (i) a thickener effect that allows the incorporation of the MOFs, without separation or loss of the filler during preparation, and (ii) induction of the acidity required for dissolving chitosan, avoiding its previous dissolution in acetic acid, thus allowing only one mixing step. Additionally, the addition of lactic acid improves the elastic performance of the final material, resulting in a large increase in the Young’s modulus and preserving the macroscopic identity of the sponge when immersed in the solution (avoiding its dissolution after swelling).

The large removal of MO in sponges loaded with MIL-100(Fe), about 95% after about 30 min of exposition in batch and flow, indicates the high potential of this MOF as a filler in bio-based lyophilized sponges. For instance, in the case of MB the base sponge does not remove the dye, but the incorporation of MIL-100(Fe) promotes its removal up to 40%. Remarkably, the other MOF, ZIF-8, does not promote the sorption of MB, and MO is removed at a lower percentage than in the case of MIL-100(Fe), indicating that specific MOF–dye interactions must play a central role, possibly related to the presence of mesoporous cages in MIL-100(Fe).

The experimental observation that the anionic base sponge does not remove the cationic MB highlights that the physical chemistry of the sorption process is complex and cannot be interpreted solely based on net charge–charge interactions. For instance, the removal percentage of MB is enhanced by adding two anionic fillers, bentonite (removal above 90%) and MIL-100(Fe), but not in the case of the also anionic celite 545 and not in sponges loaded with the cationic ionenes at low or moderate ionic strengths (below 10^−1^ M), with similar results under batch or flow conditions. This suggests that systematic studies changing the ionic strength (I) in a broad range can be appropriate for further comprehension of the sorption dynamics in the lyophilized sponges, since I may modulate surface charges and swelling effects. The swelling of sponges, facilitating the entry of the dye–aqueous solution into internal regions of the sponge, plays a central role, both in the equilibrium and kinetic aspects. We have observed that swelling reduction by increasing the ionic strength produces several effects by compacting the polymeric structure: stabilization of the structure, slowing down the removal kinetics, and promoting adsorption in the most external–superficial regions of the sponge.

The experimental kinetic results cannot be satisfactorily described by either first- or second-order kinetics in any of the considered sponges. Therefore, the Langmuir kinetics model, originally developed for adsorption–desorption processes in surface chemistry, was applied to processes where swelling is relevant and allows the solution to enter into the matrix and the dyes to be absorbed inside, like in the present situation, obtaining excellent fits in all cases. These results confirm that a more complex model, beyond pseudo-first- and second-order kinetics, is needed to describe the uptake of the dyes in the lyophilized sponges, which was successfully described here based on a rigorous formalism. These studies indicate that the removal kinetics in the aerogels must be modeled by explicitly including sorption and desorption processes. These considerations must be taken into account when designing bio-based sponges loaded with MOFs for environmental processes.

## Figures and Tables

**Table 1:** Recovered fitting parameters for the sorption kinetics of MO in sponges with different fillers, adjusted by Langmuir kinetics. The values are the results of fitting the data of Figure 7. T = (296 ± 2) K; pH = (5.5 ± 0.5).

Filler	Langmuir Kinetics for MO Removal (Equation (10))			
	$k_{1}\times 10^{2}$ (min^−1^)	$k_{2}\times 10^{2}$ (g mg^−1^ min^−1^)	$k_{s}$/1000 (M^−1^ min^−1^)	*R* ^2^
MIL-100(Fe)	3.4 ± 0.5	7 ± 2	8 ± 2	0.9998
ZIF-8	3.8 ± 0.4	2.6 ± 0.5	2.8 ± 0.5	0.9997
Bentonite	3.7 ± 0.4	3.0 ± 0.5	3.3 ± 0.5	0.9997
Celite 545	4.2 ± 0.5	8 ± 3	9 ± 3	0.9996
Base Sponge	3.6 ± 0.8	5 ± 2	5 ± 2	0.9990
Ionene 3,3′	2 ± 1	9 ± 7	10 ± 8	0.9974
Ionene 6,6′	3 ± 1	6 ± 3	7 ± 3	0.9964

*V* = 10 mL, *M_s_* = 30 mg, *X* = 9.2 × 10^−3^ mol L^−1^, and $k_{s}$ is calculated using Equation (9b). *k_d_ ~* 0.1 min^−1^ for MO. The errors on $k_{1}$ and $k_{2}$ are recovered from fits. The errors on $k_{s}\;$ are obtained by propagation on Equation (9b).

**Table 2:** Recovered fitting parameters (Langmuir model) for the sorption kinetics of MB in sponges with different fillers (Figure 8 and Figure 9). T = (296 ± 2) K; pH = (5.5 ± 0.5).

	Langmuir Kinetics for MB Removal (Equation (10))			
Filler	$k_{1}$**×** 10^2^ (min^−1^)	$k_{2}$**×** 10^2^ (g mg^−1^min^−1^)	$k_{s}$/1000 (M^−1^ min^−1^)	*R* ^2^
MIL-100(Fe)	4.6 ± 0.8	7 ± 2	8 ± 2	0.9986
Bentonite	2.1 ± 0.9	10 ± 3	11 ± 3	0.9984

*M_s_* = 30 mg, **C_o_** = 15 ppm, *V* = 10 mL, *X* = 9.4 × 10^−3^ mol L^−1^, and $k_{s}$ is calculated using Equation (9b). *k_d_* ~ 0.1 min^−1^ is obtained for MB. The errors on $k_{1}$ and $k_{2}$ are recovered from fits. The errors on $k_{s}$ are obtained by propagation on Equation (9b).

**Table 3:** Determination coefficients, *R*^2^, obtained for the fits of kinetic data by first-order, second-order, and Langmuir kinetics for the sponges loaded with the different fillers. T = (296 ± 2) K; pH = (5.5 ± 0.5).

Filler	Dye	First-Order Kinetics	Second-Order Kinetics	Langmuir Kinetics
MIL-100(Fe)	MO	0.8727	0.9694	0.9998
ZIF-8		0.9720	0.5836	0.9997
Bentonite		0.9554	0.8373	0.9997
Celite		0.8378	0.9962	0.9996
Base sponge		0.9766	0.7764	0.9990
Ionene 3,3′		0.9105	0.9897	0.9974
Ionen 6,6′		0.9629	0.9729	0.9964
MIL-100(Fe)	MB	0.9602	0.9739	0.9986
Bentonite		0.9660	0.7986	0.9984

**Table 4:** Comparison with reported values of sorption parameters for MO and MB (T ≅ 298 K, pH ≅ 6).

Reference	Dye	Matrix	Filler	*k*_2_ or *k*_2*nd*_ (gmg^−1^min^−1^)	*q_max_* (mg g^−1^)	*K_L_* (Lmg^−1^)
This work	MO	Chitosan–pectin– lactic acid	MIL-100(Fe)	0.07 (*k*_2_)	53	0.15
Zeng et al. [32]		Chitosan	Rectorite–Fe_3_O_4_-	0.02 (*k*_2*nd*_)	6	0.1
Bahrudin et al. [30]		Chitosan	Montmorinollite	0.02 (*k*_2*nd*_)	25	0.02
This work	MB	Chitosan–pectin– lactic acid	Bentonite	0.02 (*k*_2_)	21	0.2
Fan et al. [35]		Chitosan	Graphene oxide–Fe_3_O_4_	0.1 (*k*_2*nd*_)	50	0.3
Zeng et al. [32]		Chitosan-	Rectorite–Fe_3_O_4_	0.03 (*k*_2*nd*_)	25	0.2
Albadarian et al. [31]		Chitosan–lignin	-	0.2 (*k*_2*nd*_)	36	-
Lee et al. [70]		Alginate	Celite 545	0.02 (*k*_2*nd*_)	28	0.03

## Data Availability

No new data were created.

## Supplementary Materials

The following supporting information can be downloaded at https://www.mdpi.com/article/10.3390/polym17152008/s1: Figure S1. Data of the synthesized MIL-100(Fe). (a) PXRD diffractogram. (b) N_2_(g)-adsorption-desorption isotherms at 298 K. (c) SEM image. Figure S2. (a) Details of the protocol for obtaining lyophilized sponges. On the left: pectin is dissolved in DI-water at 65 °C, then, lactic acid, the filler, and chitosan are added in this order at room temperature, RT (≅21–25 °C). On the center: the formed hydrogels are placed in wells (diameter: 2.5 cm, height: 2.0 cm). On the right: composite sponges obtained after water elimination by freeze-drying (lyophilized sponges). Figures (b–d): chemical structures of the main organic components. Figure S3. Differential scanning calorimetry (DSC plots). (a) Chitosan powder. (b) Pectin powder. (c) Base sponge. (d) Sponge loaded with MIL-100(Fe). The plots correspond to the second heating cycle. Figure S4. Thermogravimetric analysis (TGA) of the lyophilized sponges loaded with (a) MIL-100(Fe), (b) ZIF-8, (c) bentonite, (d) celite 545, (e) ionene 3,3 and (f) ionene 6,6. The results of the base sponge (without adding fillers) are presented for comparison. Figure S5. Details of dye removal. Figures (a–c): Pictures of the initial MO solution (**C_o_** = 45 ppm), after 30 min of contact with a base sponge, and after 30 min of contact with a sponge containing MIL-100(Fe), respectively. Figures (d–f): analogous results for MB (**C_0_** = 15 ppm) Figures (g,h): visible-absorption spectra of the indicated MO and MB solutions, before and after contact with the sponges. In (a–h): *M_s_* = 30 mg; contact time: 30 min; *V* =10 mL. Figures (i,j): **q_e_** vs. **C_o_** (initial dye concentration) and **q_e_** vs. **C_e_** (equilibrium dye concentration), respectively, in the cases of the more efficient sponges for each dye (the fillers and dyes are indicated in the figures; *M_s_* = 30 mg; *V* =10 mL). T = (296 ± 2) K; pH = (5.5 ± 0.5). Figure S6. Removal percentage for MO with a sponge loaded with MIL-100(Fe) (10 % weight) in successive cycles of sorption-desorption. After sorption, MO is desorbed with an acid solution (pH = 4). *M_s_* = 30 mg; *V* =10 mL; **C_0_** = 45 and 15 ppm, for MO and MB, respectively. T = (296 ± 2) K; pH = (5.5 ± 0.5).

## References

[1] Nour A., Iqbal W., Navarro-Alapont J., Ferrando-Soria J., Magarò P., Elliani R., Tagarelli A., Maletta C., Mastropietro T.F., Pardo E. (2024). Efficient Nickel and Cobalt Recovery by Metal–Organic Framework-Based Mixed Matrix Membranes (MMM-MOFs). ACS Sustain. Chem. Eng..

[2] Keshta B.E., Yu H., Wang L., Gemeay A.H. (2024). Cutting-edge in the green synthesis of MIL-101(Cr) MOF based on organic and inorganic waste recycling with extraordinary removal for anionic dye. Sep. Purif. Technol..

[3] Yu L., Ji J., Chen R., Jia Q. (2024). Stable Superhydrophobic MOF-5@polydopamine@melamine Sponge for Efficient Continuous Oil–Water Separation and Emulsion Purification. ACS Appl. Polym. Mater..

[4] Sosa M.D., Alvares C.M.S., Soteras T., Levy I.K., Semino R., Negri R.M. (2024). Preparation and Comparison of Merit Parameters of Hydrophobic Membranes on Metal Meshes based on ZIF-8/PVDF and in situ Formation of ZIF-8 by Secondary Growth and Electrochemical Synthesis. ACS Appl. Polym. Mater..

[5] Pan S.-Y., Hsiao Y.-Y., Negi S., Matsagar B.M., Wu K.C.-W. (2024). Green Synthesis of Waste-Derived Metal–Organic Frameworks for Organic Substance Extraction from Piggery Wastewater as Biofertilizers. ACS Sustain. Chem. Eng..

[6] Kaur H., Devi N., Siwal S.S., Alsanie W.F., Thakur M.K., Thakur V.K. (2023). Metal–Organic Framework-Based Materials for Wastewater Treatment: Superior Adsorbent Materials for the Removal of Hazardous Pollutants. ACS Omega.

[7] Karami K., Beram S.M., Bayat P., Siadatnasab F., Ramezanpour A. (2022). A novel nanohybrid based on metal–organic framework MIL101−Cr/PANI/Ag for the adsorption of cationic methylene blue dye from aqueous solution. J. Mol. Struct..

[8] Freund R., Zaremba O., Arnauts G., Ameloot R., Skorupskii G., Dincă M., Bavykina A., Gascon J., Ejsmont A., Goscianska J. (2021). The Current Status of MOF and COF Applications. Angew. Chem. Int. Ed. Engl..

[9] Yu S., Pang H., Huang S., Tang H., Wang S., Qiu M., Chen Z., Yang H., Song G., Fu D. (2021). Recent advances in metal-organic framework membranes for water treatment: A review. Sci. Total. Environ..

[10] Parmar B., Bisht K.K., Rajput G., Suresh E. (2021). Recent advances in metal–organic frameworks as adsorbent materials for hazardous dye molecules. Dalton Trans..

[11] Lu H., Liu B., Huang H., Zou C., Tang L., Liu J., Wang C., Liang J. (2025). Efficient elimination of organic contaminants via a non-radical pathway involving activation of sulfite by synergistic adsorption-catalysis bifunctional chitosan-modified MOF derivative. Sep. Purif. Technol..

[12] Salazar H., Rosales M., Zarandona I., Serra J., Gonçalves B.F., Valverde A., Cavalcanti L.P., Lanceros-Mendez S., García A., de la Caba K. (2024). Metal-Organic Framework Functionalized Chitosan/Pectin Membranes for Solar-Driven Photo-Oxidation and Adsorption of Arsenic. Chem. Eng. J..

[13] Zhang Y., Yuan H., Chen X., Jiang Z., Lu J., Xin F. (2024). Incorporating MXene@MOF-303 Composites into Poly(vinyl alcohol) (PVA) to Fabricate Pervaporation Membranes for Desalination. ACS Appl. Polym. Mater..

[14] Zhang A., Shan F., Zhang Z., Wang J., Zhang T., Liu M. (2024). Efficient decontamination of tetracycline via fenton-like process mediated by chitosan-based Fe-MOFs under wide pH range. Sep. Purif. Technol..

[15] Duan J., Li Q., Xu W., Hu X., Wang Y., Valdez S.M., Qiang Z., Liao Y., Wen J., Ye C. (2024). Mechanically Flexible and Weavable Hybrid Aerogel Fibers with Ultrahigh Metal–Organic Framework Loadings for Versatile Applications. ACS Appl. Polym. Mater..

[16] Wang A., Ni J., Wang W., Liu D., Zhu Q., Xue B., Chang C.-C., Ma J., Zhao Y. (2022). MOF Derived Co−Fe nitrogen doped graphite carbon@crosslinked magnetic chitosan Micro−nanoreactor for environmental applications: Synergy enhancement effect of adsorption−PMS activation. Appl. Catal. B Environ..

[17] Chen M., Long A., Zhang W., Wang Z., Xiao X., Gao Y., Zhou L., Li Y., Wang J., Sun S. (2025). Recent advances in alginate-based hydrogels for the adsorption–desorption of heavy metal ions from water: A review. Sep. Purif. Technol..

[18] Li J., Lin G., Liang H., Wang S., Hu T., Zhang L. (2025). Adsorptive removal of Pb(II) using magnetic MOFs-modified chitosan composite: Preparation, performance and mechanism. Sep. Purif. Technol..

[19] Xue T., Wang M., Man J., Yang Y., Miao H., Li X. (2024). Construction and Regulation of a Superhydrophobic Sponge via In Situ Anchoring of a Hyper-Cross-Linked Polymer for Efficient Oil/Water Separation. ACS Appl. Polym. Mater..

[20] Striz R., Minisy I.M., Bober P., Taboubi O., Smilek J., Kovalcik A. (2024). Free-Standing Bacterial Cellulose/Polypyrrole Composites for Eco-Friendly Remediation of Hexavalent Chromium Ions. ACS Appl. Polym. Mater..

[21] Wang X., Feng X., Li Q., Dong Z. (2024). Surface Functionalization Strategy for Cellulose Membranes Based on Silanization and Thiol–Ene Click Chemistry. ACS Appl. Polym. Mater..

[22] Zhang X., Liu M., Zhang C., Yuan Z., Chi H. (2024). Real-Time Uranyl Ion Adsorption Monitoring Based on Cellulose Hydrogels. ACS Appl. Polym. Mater..

[23] Ahmadian M., Jaymand M. (2023). Interpenetrating polymer network hydrogels for removal of synthetic dyes: A comprehensive review. Coord. Chem. Rev..

[24] Pandey S., Makhado E., Kim S., Kang M. (2023). Recent developments of polysaccharide based superabsorbent nanocomposite for organic dye contamination removal from wastewater—A review. Environ. Res..

[25] Rodríguez-Ramírez C., Tasqué J.E., Garcia N.L., D’ACcorso N.B. (2023). Hemicelluloses hydrogel: Synthesis, characterization, and application in dye removal. Int. J. Biol. Macromol..

[26] Sivakumar R., Lee N.Y. (2022). Adsorptive removal of organic pollutant methylene blue using polysaccharide-based composite hydrogels. Chemosphere.

[27] Chen T., Liu H., Gao J., Hu G., Zhao Y., Tang X., Han X. (2022). Efficient Removal of Methylene Blue by Bio-Based Sodium Alginate/Lignin Composite Hydrogel Beads. Polymers.

[28] Sirajudheen P., Poovathumkuzhi N.C., Vigneshwaran S., Chelaveettil B.M., Meenakshi S. (2021). Applications of chitin and chitosan based biomaterials for the adsorptive removal of textile dyes from water—A comprehensive review. Carbohydr. Polym..

[29] Roa K., Tapiero Y., Thotiyl M.O., Sánchez J. (2021). Hydrogels Based on Poly([2-(acryloxy)ethyl] Trimethylammonium Chloride) and Nanocellulose Applied to Remove Methyl Orange Dye from Water. Polymers.

[30] Bahrudin N.N., Nawi M.A., Jawad A.H., Sabar S. (2020). Adsorption Characteristics and Mechanistic Study of Immobilized Chitosan-Montmorillonite Composite for Methyl Orange removal. J. Polym. Environ..

[31] Albadarin A.B., Collins M.N., Naushad M., Shirazian S., Walker G., Mangwandi C. (2017). Activated lignin-chitosan extruded blends for efficient adsorption of methylene blue. Chem. Eng. J..

[32] Zeng L., Xie M., Zhang Q., Kang Y., Guo X., Xiao H., Peng Y., Luo J. (2015). Chitosan/organic rectorite composite for the magnetic uptake of methylene blue and methyl orange. Carbohydr. Polym..

[33] Auta M., Hameed B. (2014). Chitosan–clay composite as highly effective and low-cost adsorbent for batch and fixed-bed adsorption of methylene blue. Chem. Eng. J..

[34] Zhang J., Zhou Q., Ou L. (2011). Kinetic, Isotherm, and Thermodynamic Studies of the Adsorption of Methyl Orange from Aqueous Solution by Chitosan/Alumina Composite. J. Chem. Eng. Data.

[35] Fan L., Luo C., Li X., Lu F., Qiu H., Sun M. (2012). Fabrication of novel magnetic chitosan grafted with graphene oxide to enhance adsorption properties for methyl blue. J. Hazard. Mater..

[36] Khan M.A., Kuldeep, Yadav S., Singh N., Basheed G. (2025). Enhanced adsorption of congo red dye using dried chitosan functionalized MnFe2O4 viscoelastic fluid. Colloids Surf. A Physicochem. Eng. Asp..

[37] Foroutan R., Tutunchi A., Foroughi M., Ramavandi B. (2025). Efficient fluoride removal from water and industrial wastewater using magnetic chitosan/β-cyclodextrin aerogel enhanced with biochar and MOF composites. Sep. Purif. Technol..

[38] Liu C., Zhao M., Liu H., Zhang J., Hu Z., Zhang Y., Pang X. (2024). Robust and anti-biofouling bio-based aerogel with Schiff base network stabilized MOFs for efficient removal of tartrazine dye and U(VI) ions. Sep. Purif. Technol..

[39] Dubovenko R., Kuzminova A., Dmitrenko M., Stepanova A., Selyutin A., Su R., Penkova A. (2024). Enhanced Sodium Alginate Membranes Modified with Metal–Organic Frameworks Based on Zirconium for Energy-Efficient Isopropanol Dehydration by Pervaporation. ACS Appl. Polym. Mater..

[40] Zhu L., Lu H., Huo T., Liu D., Yan Z., Zhang J. (2025). Superhydrophobic PDMS/MOF-74@PU sponge with photothermal property for efficient oil/water separation. Sep. Purif. Technol..

[41] Bera P., Mukherjee S., Venturi D.M., Ruser N., Biswas S. (2024). Reusable MOF-Coated Chitosan@Paper Strip Composite for Real-Time Monitoring of Pesticide Pendimethalin and Organoarsenic Feed Additive Roxarsone Levels in Environmental Water, Food, and Vegetable Samples. ACS Appl. Mater. Interfaces.

[42] Sacourbaravi R., Ansari-Asl Z., Hoveizi E., Darabpour E. (2024). Poly(vinyl alcohol)/Chitosan Hydrogel Containing Gallic Acid-Modified Fe, Cu, and Zn Metal–Organic Frameworks (MOFs): Preparation, Characterization, and Biological Applications. ACS Appl. Mater. Interfaces.

[43] Ghosh S., Mal D., Mukherjee S., Biswas S. (2023). Sustainable Fabrication of an Eco-Friendly, Reusable Chitosan@Cotton@MOF Composite Sensor for 2,4-Dichlorophenoxyacetic Acid Herbicide and Nitroxoline Antibiotic. ACS Sustain. Chem. Eng..

[44] Valadi F.M., Shahsavari S., Akbarzadeh E., Gholami M.R. (2022). Preparation of new MOF-808/chitosan composite for Cr(VI) adsorption from aqueous solution: Experimental and DFT study. Carbohydr. Polym..

[45] Karakaş A., Topçu E., Erçarıkcı E., Kıranşan K.D. (2025). MOF-containing graphene sponge for efficient solar desalination and water purification. Sep. Purif. Technol..

[46] Tran D.T., Lei L., Song M.-H., Cui L., Mao J., Lin X., Yun Y.-S. (2025). Polyethyleneimine-functionalized pectin fibers as effective adsorbents for the removal of mercury ions from aqueous solution: Characterization, performance, and mechanism. Sep. Purif. Technol..

[47] Yang Q., Zhao Z., Liu R., Yan Z., Yu J., Chen L., Li X., Cao C., Yao F., Zhang H. (2024). Water-Triggered Self-Expanding Agarose/Chitosan-Gallate Hemostatic Sponge for Incompressible Wounds. ACS Appl. Polym. Mater..

[48] Wu Y., Yan Z., Wang T., Wang J., Wang T., Hu Z., Ao Y., Wang Y., Li M. (2023). Cellulose Nanofibers/PEDOT:PSS Conductive Aerogel for Pressure Sensing Prepared by a Facile Freeze-Drying Method. ACS Appl. Polym. Mater..

[49] Liu J., Xie X., Wang T., Chen H., Fu Y., Cheng X., Wu J., Li G., Liu C., Liimatainen H. (2023). Promotion of Wound Healing Using Nanoporous Silk Fibroin Sponges. ACS Appl. Mater. Interfaces.

[50] Lee J., Choi H.N., Cha H.J., Yang Y.J. (2023). Microporous Hemostatic Sponge Based on Silk Fibroin and Starch with Increased Structural Retentivity for Contact Activation of the Coagulation Cascade. Biomacromolecules.

[51] Qi X., Gan J., Zhao Z., Li N., Chen Y., Jin T. (2023). Chitosan Sponge/Cu–WO_3–*x*_ Composite for Photodynamic Therapy of Wound Infection. Langmuir.

[52] Ma Y., You D., Fang Y., Luo J., Pan Q., Liu Y., Wang F., Yang W. (2022). Confined growth of MOF in chitosan matrix for removal of trace Pb(II) from reclaimed water. Sep. Purif. Technol..

[53] He P., Gu G., Xu Y., Wei G., Xu M. (2025). Injectable and self-healing dual-network chitosan/hyaluronic acid/polypeptide (CHP) antibacterial hydrogels for wound healing. Polymer.

[54] Xue X., Miao X., Liu J., Ding Y., Zhang Y., Sun Y., Huang W., Jiang Q., Jiang B., Komarneni S. (2025). Investigating the pH-Dependence of gelation process in chitosan-glutaraldehyde hydrogels with diffusing wave spectroscopy. Polymer.

[55] Luo X., Wang C., Huang G., Tan Y., Tang W., Kong J., Li Z. (2022). Bio-inspired chitosan aerogel decorated with MOF-on-COF heterostructure hybrid as recyclable scavenger of herbicides in water. Sep. Purif. Technol..

[56] Sam E.K., Liu J., Lv X. (2021). Surface Engineering Materials of Superhydrophobic Sponges for Oil/Water Separation: A Review. Ind. Eng. Chem. Res..

[57] Zhang X., Xu Z., Li K., Li X., Deng S., Liu Y., Zhu G. (2024). Porous Cu-BTC Metal–Organic Frameworks Anchored on Dialdehyde Wood Sponge as Material for CO_2_ Capture and Separation. ACS Appl. Nano Mater..

[58] Sun X., Yu Q., Wang F., Sun M., Hu S., Zhu J., Li C., Yang Z., Liu Y., Zhou J. (2024). Eco-Friendly Tourmaline@MOF Lignocellulose Aerogel with Favorable Fire Retardancy and Smoke Suppression for Insulation Materials. ACS Sustain. Chem. Eng..

[59] Cao K., Yang X., Zhao R., Xue W. (2023). Fabrication of an Ultralight Ni-MOF-rGO Aerogel with Both Dielectric and Magnetic Performances for Enhanced Microwave Absorption: Microspheres with Hollow Structure Grow onto the GO Nanosheets. ACS Appl. Mater. Interfaces.

[60] Peng X., Zhang J., Sun J., Liu X., Zhao X., Yu S., Yuan Z., Liu S., Yi X. (2023). Hierarchically Porous Mg-MOF-74/Sodium Alginate Composite Aerogel for CO_2_ Capture. ACS Appl. Nano Mater..

[61] Habibi N., Faraji S., Pourjavadi A. (2023). Nano graphite platelets/Cu (BDC) MOF coating on polyurethane sponge: A superhydrophobic self-extinguishing adsorbent for static and continuous oil/water separation. Colloids Surf. A Physicochem. Eng. Asp..

[62] He Z., Wu H., Shi Z., Duan X., Ma S., Chen J., Kong Z., Chen A., Sun Y., Liu X. (2022). Mussel-inspired durable superhydrophobic/superoleophilic MOF-PU sponge with high chemical stability, efficient oil/water separation and excellent anti-icing properties. Colloids Surf. A Physicochem. Eng. Asp..

[63] Jeyaseelan A., Viswanathan N., Naushad M. (2025). Design and development of rare earth elements anchored pectin/chitosan integrated magnesia hybrid composite for effective defluoridation of water. Sep. Purif. Technol..

[64] Kloster M., Marcovich N.E., Mosiewicki M.A. (2024). Microcrystalline cellulose modified chitosan aerogels to enhance Congo Red dye adsorption. Colloids Surf. A Physicochem. Eng. Asp..

[65] Rajendran J., Panneerselvam A., Ramasamy S., Palanisamy P. (2024). Methylene blue and methyl orange removal from wastewater by magnetic adsorbent based on activated carbon synthesised from watermelon shell. Desalination Water Treat..

[66] Kloster M., Mosiewicki M.A., Marcovich N.E. (2024). Removal of dyes from aqueous media using environmentally friendly aerogels based on chitosan. Colloids Surf. A Physicochem. Eng. Asp..

[67] İpek Ö., Taşar Ş., Duranay N. (2025). Removal of basic yellow dye molecules with chitosan-based magnetic field-sensitive particles from the aqueous solution. ACS Polymer.

[68] Rahul, Jindal R. (2024). Efficient removal of toxic dyes malachite green and fuchsin acid from aqueous solutions using Pullulan/CMC hydrogel. ACS Polymer.

[69] Dong Y., Abbasi A., Mohammadnejad S., Nasrollahzadeh M., Sheibani R., Otadi M. (2024). Recent progresses in bentonite/lignin or polysaccharide composites for sustainable water treatment. Int. J. Biol. Macromol..

[70] Jabli M., Almalki S.G., Agougui H. (2020). An insight into methylene blue adsorption characteristics onto functionalized alginate bio-polymer gel beads with λ-carrageenan-calcium phosphate, carboxymethyl cellulose, and celite 545. Int. J. Biol. Macromol..

[71] Lee J.S., Hocken A., Green M.D. (2021). Advances in the molecular design of ionenes for a diverse range of applications. Mol. Syst. Des. Eng..

[72] Hotton C., Ducouret G., Sirieix-Plénet J., Bizien T., Porcar L., Malikova N. (2023). Tuning Structure and Rheological Properties of Polyelectrolyte-Based Hydrogels through Counterion-Specific Effects. Macromolecules.

[73] Malikova N., Rollet A.-L., Čebašek S., Tomšič M., Vlachy V. (2015). On the crossroads of current polyelectrolyte theory and counterion-specific effects. Phys. Chem. Chem. Phys..

[74] Tanaka S., Tanaka Y. (2019). A Simple Step toward Enhancing Hydrothermal Stability of ZIF-8. ACS Omega.

[75] Khan I.U., Othman M.H.D., Jilani A., Ismail A., Hashim H., Jaafar J., Rahman M.A., Rehman G.U. (2018). Economical, environmental friendly synthesis, characterization for the production of zeolitic imidazolate framework-8 (ZIF-8) nanoparticles with enhanced CO2 adsorption. Arab. J. Chem..

[76] James J.B., Lin Y.S. (2016). Kinetics of ZIF-8 Thermal Decomposition in Inert, Oxidizing, and Reducing Environments. J. Phys. Chem. C.

[77] Ordoñez M.J.C., Balkus K.J., Ferraris J.P., Musselman I.H. (2010). Molecular sieving realized with ZIF-8/Matrimid® mixed-matrix membranes. J. Membr. Sci..

[78] Simon M.A., Anggraeni E., Soetaredjo F.E., Santoso S.P., Irawaty W., Thanh T.C., Hartono S.B., Yuliana M., Ismadji S. (2019). Hydrothermal Synthesize of HF-Free MIL-100(Fe) for Isoniazid-Drug Delivery. Sci. Rep..

[79] Zhang F., Shi J., Jin Y., Fu Y., Zhong Y., Zhu W. (2015). Facile synthesis of MIL-100(Fe) under HF-free conditions and its application in the acetalization of aldehydes with diols. Chem. Eng. J..

[80] Monge M.E., Negri R.M., Kolender A.A., Erra-Balsells R. (2007). Structural Characterization of Native High-Methoxylated Pectin using NMR Spectroscopy and UV-MALDI-TOF Mass Spectrometry. Comparative use of 2,5-Dihydroxybenzoic Acid and n-Harmane as UV-MALDI Matrices. Rapid Commun. Mass Spectrom..

[81] Dutta J. (2022). Priyanka A facile approach for the determination of degree of deacetylation of chitosan using acid-base titration. Heliyon.

[82] Kasaai M.R. (2007). Calculation of Mark–Houwink–Sakurada (MHS) equation viscometric constants for chitosan in any solvent–temperature system using experimental reported viscometric constants data. Carbohydr. Polym..

[83] Baik M.H., Lee S.Y. (2010). Colloidal stability of bentonite clay considering surface charge properties as a function of pH and ionic strength. J. Ind. Eng. Chem..

[84] Alves F.F.C., Morais A.Í.S., Lima L.C.B., Santos A.M.S., Lima I.S., Silva A.S., Garcia R.R.P., Braga A.N.S., Cuevas M.D.M.O., Carrasco S.M. (2024). A new composite based on gellan gum/chitosan and hydroxyapatite contains gallium for removing the anionic dyes remazol blue and remazol red. J. Polym. Environ..

[85] Ben Seghir B., Benhamza M.H. (2017). Preparation, optimization and characterization of chitosan polymer from shrimp shells. J. Food Meas. Charact..

[86] Sousa J.M., Vieira A.C., Costa M.P., Rizzo M.S., Chaves L.L., Braz E.M., Bezerra R.D., Leal R.C., Barreto H.M., Osajima J.A. (2022). Chitosan grafted with maleic anhydride and ethylenediamine: Preparation, characterization, computational study, antibacterial and cytotoxic properties. Mater. Chem. Phys..

[87] Marudova M., MacDougall A.J., Ring S.G. (2004). Pectin–chitosan interactions and gel formation. Carbohydr. Res..

[88] Chang K. (2000). Swelling behavior and the release of protein from chitosan–pectin composite particles. Carbohydr. Polym..

[89] Yao K.D., Liu J., Cheng G.X., Lu X.D., Tu H.L., Lopes da Silva J.A. (1996). Swelling behavior of pectin/chitosan complex films. J. Appl. Polym. Sci..

[90] Hoagland P.D., Parris N. (1996). Chitosan/Pectin Laminated Films. J. Agric. Food Chem..

[91] Ralet M.-C., Dronnet V., Buchholt H.C., Thibault J.-F. (2001). Enzymatically and chemically de-esterified lime pectins: Characterisation, polyelectrolyte behaviour and calcium binding properties. Carbohydr. Res..

[92] Ström A., Schuster E., Goh S.M. (2014). Rheological characterization of acid pectin samples in the absence and presence of monovalent ions. Carbohydr. Polym..

[93] Maciel V.B.V., Yoshida C.M., Franco T.T. (2015). Chitosan/pectin polyelectrolyte complex as a pH indicator. Carbohydr. Polym..

[94] Sahebjamee N., Soltanieh M., Mousavi S.M., Heydarinasab A. (2020). Preparation and characterization of porous chitosan–based membrane with enhanced copper ion adsorption performance. React. Funct. Polym..

[95] Tan F., Liu M., Li K., Wang Y., Wang J., Guo X., Zhang G., Song C. (2015). Facile synthesis of size-controlled MIL-100(Fe) with excellent adsorption capacity for methylene blue. Chem. Eng. J..

[96] Huo S.-H., Yan X.-P. (2012). Metal–organic framework MIL-100(Fe) for the adsorption of malachite green from aqueous solution. J. Mater. Chem..

[97] Oh S., Lee S., Lee G., Oh M. (2023). Enhanced adsorption capacity of ZIF-8 for chemical warfare agent simulants caused by its morphology and surface charge. Sci. Rep..

[98] Gao M., Li L., Sun Z., Li J., Jiang H. (2022). Facet engineering of a metal–organic framework support modulates the microenvironment of palladium nanoparticles for selective hydrogenation. Angew. Chem. Int. Ed. Engl..

[99] Wang S., Ouyang L., Deng G., Deng Z., Wang S. (2020). DNA adsorption on nanoscale zeolitic imidazolate framework-8 enabling rational design of a DNA-based nanoprobe for gene detection and regulation in living cells. RSC Adv..

[100] Ding Y., Xu Y., Ding B., Li Z., Xie F., Zhang F., Wang H., Liu J., Wang X. (2017). Structure induced selective adsorption performance of ZIF-8 nanocrystals in water. Colloids Surf. A Physicochem. Eng. Asp..

[101] Hsu C.H., Tsai S.W. (2001). Improvements of Acinetobacter Radioresistens Lipase Adsorption on Celite 535 by Adding Salts. J. Appl. Eng. Sci..

[102] Oulman C.S., Baumann E.R. (1964). Streaming Potentials in Diatomite Filtration of Water. J. AWWA.

[103] Ullah I., Xiang F., Li Y., Huang J., Ans M., Iqbal J., Zhang Z., Xiang S., Khan E. (2023). Crystal Structure, Spectroscopic Studies and Supramolecular Chemistry, DFT Based Electronic and Optical Properties of Salts of Methylene Blue with Tetrahedral Anions. ChemistrySelect.

[104] You K., Kwon O., Kim D. (2023). Effects of the protonation and the polar solvation on the molecular properties of methyl orange: A density functional theory study. Bull. Korean Chem. Soc..

[105] Şenol Z.M., Ertap H., Fernine Y., El Messaoudi N. (2024). Adsorptive removal of synthetic dye from its aqueous solution by using chitosan-bentonite composite: DFT and experimental studies. Polym. Bull..

[106] Liu Y., Shen L. (2008). From Langmuir Kinetics to First- and Second-Order Rate Equations for Adsorption. Langmuir.

[107] Azizian S. (2004). Kinetic models of sorption: A theoretical analysis. J. Colloid Interface Sci..

