# Supplementary material for: Aerogels of Chitosan–Pectin–Lactic Acid Loaded with MOFs: Performance and Kinetics in Removal of Dyes
Source: Polymers (Basel). 2025 Jul 23;17(15):2008. doi: 10.3390/polym17152008 (PMC12349115; doi:10.3390/polym17152008)
Supplement: Supplementary file 1 [file polymers-17-02008-s001.zip › polymers-3746398-supplementary.pdf]

polymers

Supplementary Material. Additional figures of biopolymer sponge characterization, including DSC, TGA and PXRD.

Aerogels of Chitosan–Pectin–Lactic Acid Loaded with MOFs: Performance and Kinetics in Removal of Dyes.

Tomás Soteras†, Ignacio Manuel Argento Arruñada†, Leila María Saleh Medina, Natalie Malikova, Koro de la Caba, Pedro Guerrero*, Norma B. D´Accorso*, R. Martín Negri*.

† Considered first authors as equals. * Responsible authors.

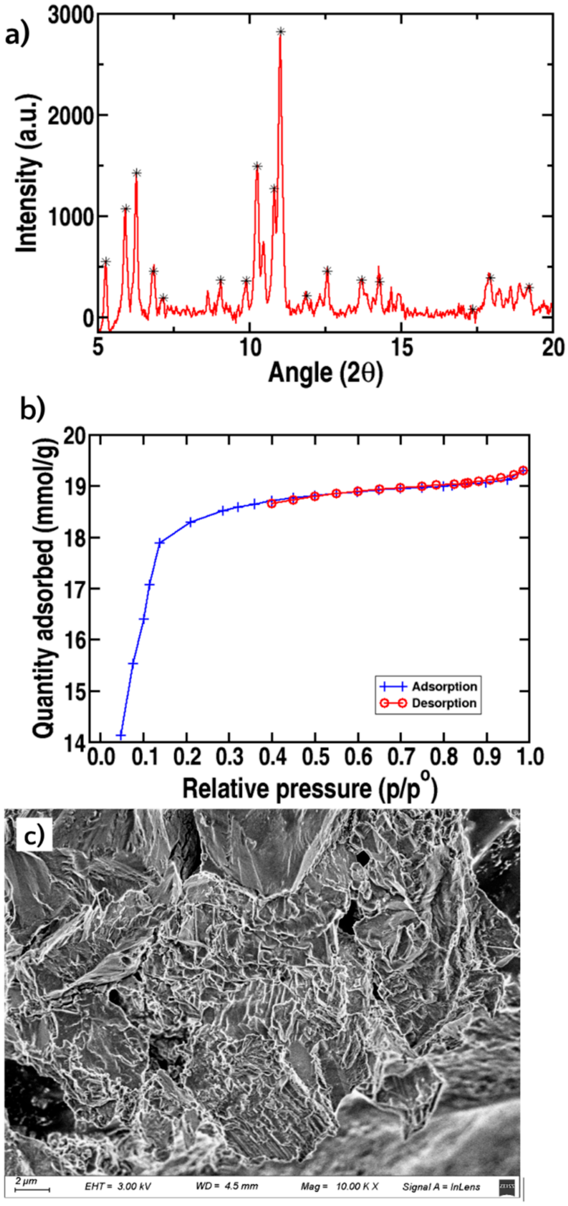

Figure S1. Data of the synthesized MIL-100(Fe). a) PXRD diffractogram. b) N_2(g) adsorption–desorption isotherms at 298 K. c) SEM image.

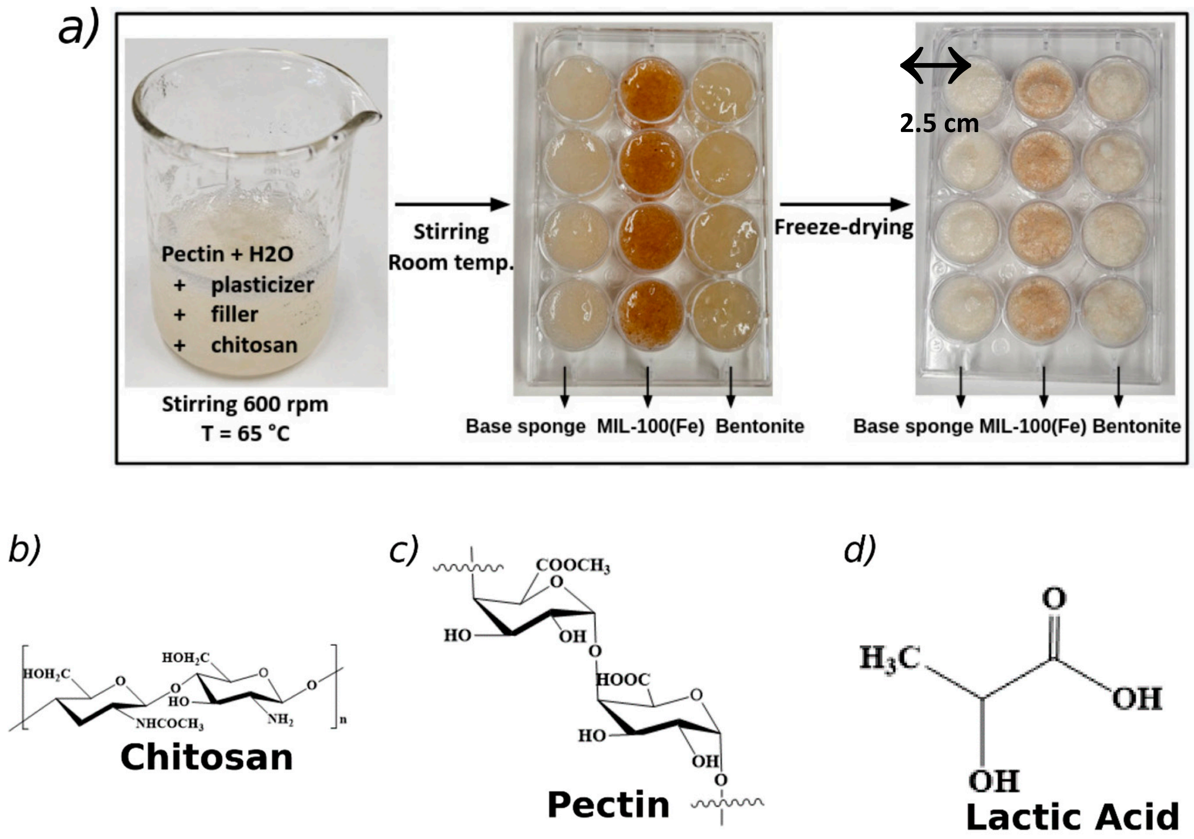

Figure S2. a) Details of the protocol for obtaining lyophilized sponges. On the left: Pectin is dissolved in DI water at 65 °C, then lactic acid, the filler, and chitosan are added in this order at room temperature, RT (≅ 21-25 °C). In the center: The formed hydrogels are placed in wells (diameter: 2.5 cm; height: 2.0 cm). On the right: Composite sponges obtained after water elimination by freeze-drying (lyophilized sponges). Figures b), c), and d): Chemical structures of the main organic components.

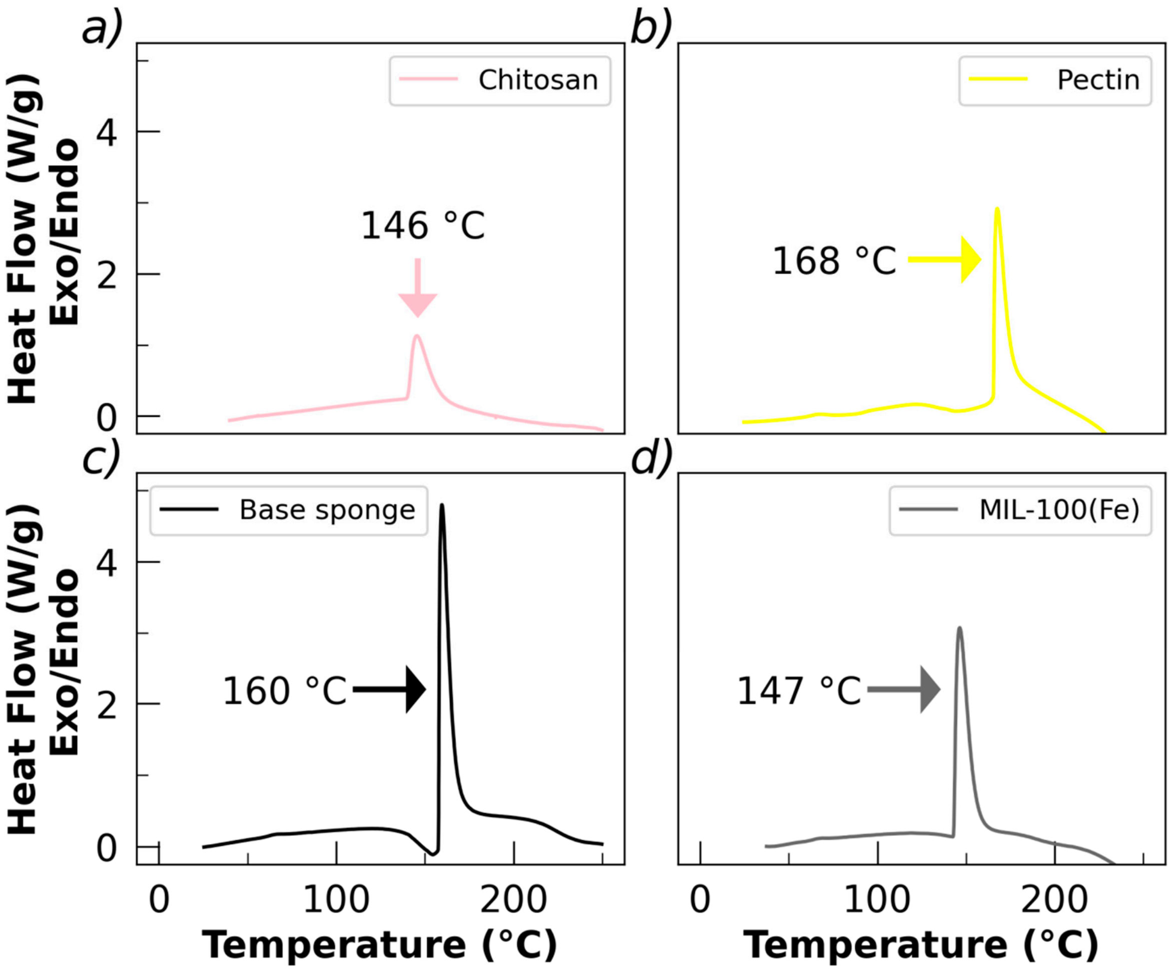

Figure S3. Differential scanning calorimetry (DSC plots). a) Chitosan powder. b) Pectin powder. c) Base sponge. d) Sponge loaded with MIL-100(Fe). The plots correspond to the second heating cycle.

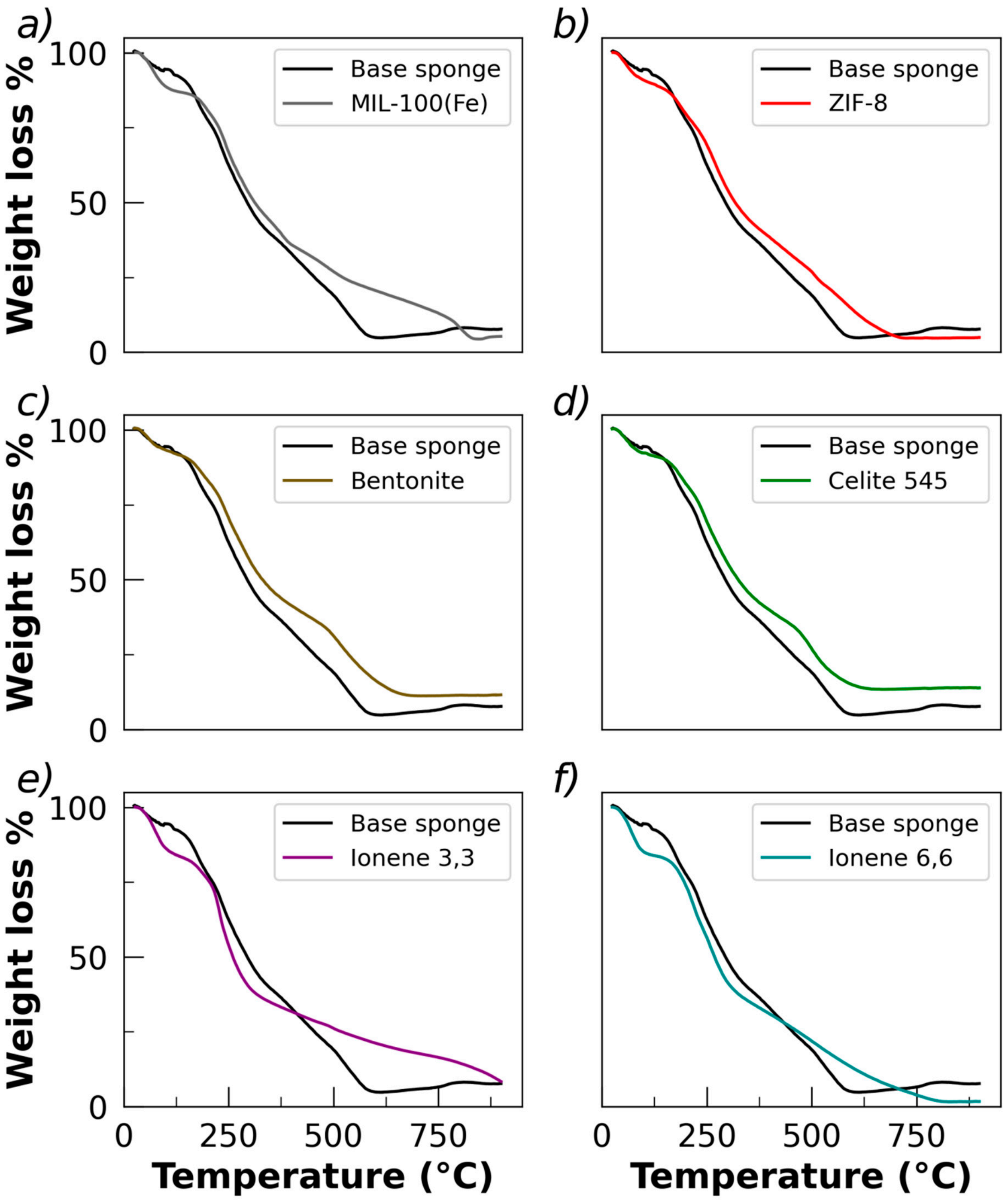

Figure S4. Thermogravimetric analysis (TGA) of the lyophilized sponges loaded with a) MIL-100(Fe), b) ZIF-8, c) bentonite, d) celite 545, e) ionene 3,3, and f) ionene 6,6. The results of the base sponge (without adding fillers) are presented for comparison.

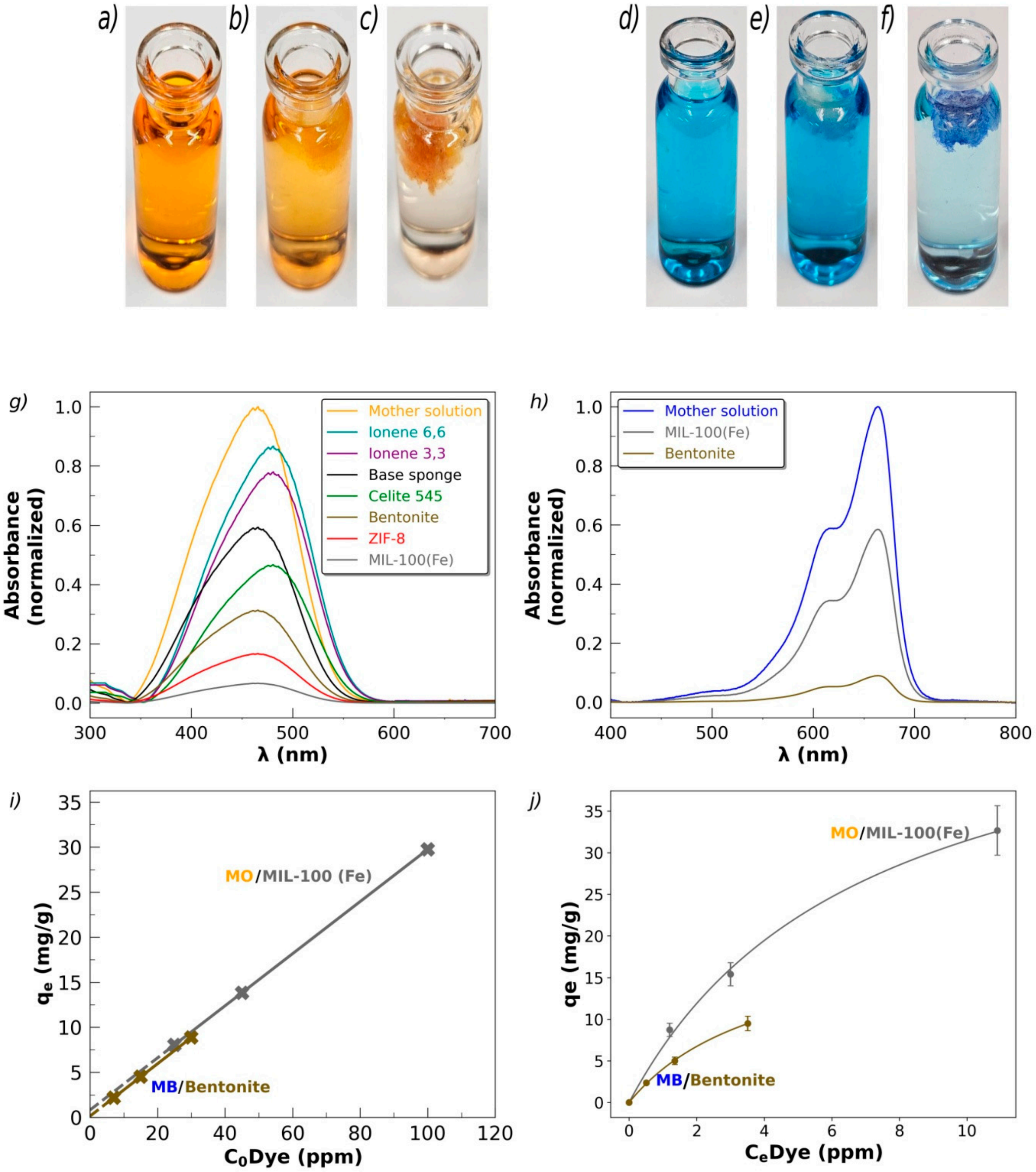

Figure S5. Details of dye removal. Figures a), b), and c): Pictures of the initial MO solution (**C_o** = 45 ppm), after 30 min of contact with a base sponge, and after 30 min of contact with a sponge containing MIL-100(Fe), respectively. Figures d), e), and f): Analogous results for MB (**C_0** = 15 ppm). Figures g) and h): Visible absorption spectra of the indicated MO and MB solutions, before and after contact with the sponges. a) to h): M_s = 30 mg; contact time: 30 min; V =10 mL. Figures i) and j): **q_e** vs. **C_o** (initial dye concentration) and **q_e** vs. **C_e** (equilibrium dye concentration), respectively, in the cases of the more efficient sponges for each dye (the fillers and dyes are indicated in the figures; M_s = 30 mg; V =10 mL). T = (296 ± 2) K; pH = (5.5 ± 0.5).

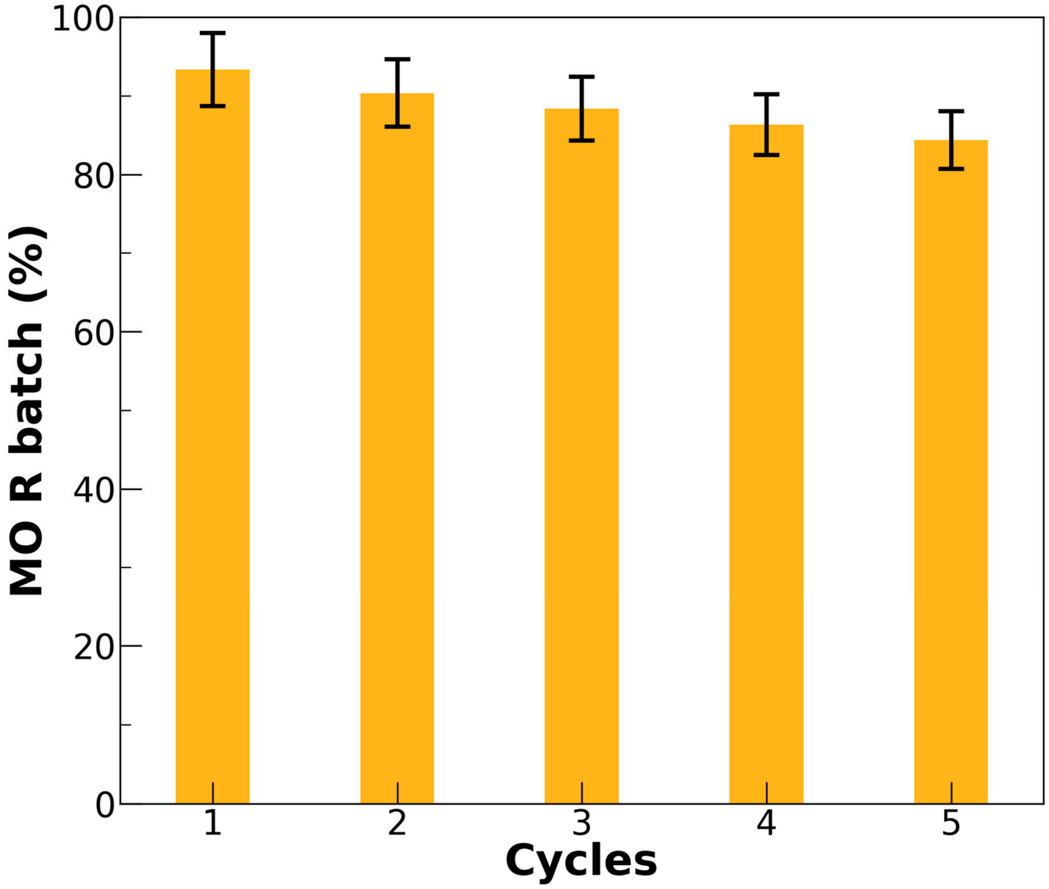

Figure S6. Removal percentage for MO with a sponge loaded with MIL-100(Fe) (10 % weight) in successive cycles of sorption–desorption. After sorption, MO is desorbed with an acid solution (pH = 4). M_s = 30 mg; V =10 mL; $\mathbf{C_0}$ = 45 and 15 ppm, for MO and MB, respectively. T = (296 ± 2) K; pH = (5.5 ± 0.5).
